# Characterisation of Elastomers as Food Contact Materials–Part 1: Quantification of Extractable Compounds, Swelling of Elastomers in Food Simulants and Release of Elements

**DOI:** 10.3390/molecules26020509

**Published:** 2021-01-19

**Authors:** Friederike Kühne, Maurus Biedermann, Angela Eicher, Florian Felder, Stefan Sander, Roman Schmidt, Saskia Lehmann, Gregor McCombie, Stefan Merkel, Oliver Kappenstein, Andreas Luch

**Affiliations:** 1 National Reference Laboratory for Food Contact Materials, Department of Chemical and Product Safety, German Federal Institute for Risk Assessment (BfR), Max-Dohrn-Str. 8-10, 10589 Berlin, Germany; Stefan.Sander@bfr.bund.de (S.S.); Roman.Schmidt@bfr.bund.de (R.S.); Saskia.Lehmann@bfr.bund.de (S.L.); Stefan.Merkel@bfr.bund.de (S.M.); Andreas.Luch@bfr.bund.de (A.L.); 2 Official Control Laboratory of the Canton of Zurich, Fehrenstr. 15, 8032 Zurich, Switzerland; Maurus.Biedermann@kl.zh.ch (M.B.); Angela.Eicher@kl.zh.ch (A.E.); florianfelder1997@gmail.com (F.F.); Gregor.McCombie@kl.zh.ch (G.M.); 3 Department of Safety in the Food Chain, German Federal Institute for Risk Assessment (BfR), Max-Dohrn-Str. 8-10, 10589 Berlin, Germany; Oliver.Kappenstein@bfr.bund.de

**Keywords:** elastomer, rubber, food contact materials, extraction, migration, elements, swelling

## Abstract

Elastomers are not a uniform class of materials but comprise a broad spectrum of chemically different polymers. Sealing gaskets, gloves, teats, conveyor belts and tubing are examples of elastomers being used as food contact materials (FCMs). Ten elastomer samples were evaluated with respect to the content of extractable compounds, migration of substances into ethanolic food simulants, swelling in food simulants and release of elements in different food simulants. The number of extractable substances <1000 Da was determined by comprehensive two-dimensional gas chromatography coupled with flame ionisation detection (GC × GC–FID) analysis of tetrahydrofuran (THF) extracts. The number of signals ranged from 61 (a thermoplastic elastomer (TPE)) to 690 (a natural rubber/styrene-butadiene-rubber blend (NR/SBR)). As for risk assessment, the decisive factor is which substances reach the food. The extent of substances that migrate into ethanolic food simulants was investigated. Elastomer FCMs can be the source of food contamination with heavy metals. Notably, contamination with lead was detected in some samples investigated in this study. It was shown that food simulants harbour the potential to morphologically alter or even disintegrate elastomeric materials. The results presented here highlight the importance to carefully choose the elastomer type for the intended use as FCMs as not every application may prove safe for consumers.

## 1. Introduction

According to the IUPAC definition, elastomers are polymers which “display rubber-like elasticity” [1]. Their use in the field of food contact materials (FCMs) is widely spread; examples are sealing gaskets, gloves, teats, conveyor belts and tubing. Elastomers encompass a wide range of chemically different polymers. Irreversibly (chemically) cross-linked elastomers which keep their elasticity up to the degradation temperature are called rubbers, which may be of natural or synthetic origin. Rubbers of the R type contain C=C double bonds and are fully or partially made of diolefins. Examples are natural rubber (NR), isobutylene-isoprene rubber (IIR), acrylonitrile butadiene rubber (NBR) and styrene-butadiene rubber (SBR). M-type rubbers, on the other hand, have saturated methylene groups as chain links, for example, ethylene propylene diene monomer rubber (EPDM). Other types of rubber are characterised by the presence of heteroatoms in the backbone and/or sidechain. The U type, for example, contains C, N and O atoms, as seen in polyurethane rubbers (PUR). In contrast to the irreversibly linked rubbers, in thermoplastic elastomers (TPE), the macromolecules are reversibly linked by physical forces, which disappear at elevated temperatures. During usage, TPEs have characteristics of elastomers while displaying a processing behaviour of thermoplastic materials. TPEs can be divided into two major groups, one being the polymer blends which consist of a hard polymer matrix with softer elastomer particles imbedded therein. The other group comprises the so-called block co-polymers consisting of hard segments and soft segments which are incompatible with each other and therefore develop (sub)microscopic segregations. The chemical diversity of TPEs is just as vast as that of rubbers [2]. Styrene-ethylene-butadiene-styrene (SEBS) and styrene-butadiene-styrene (SBS) are examples for TPE block co-polymers.

As apparent from the description above, elastomers are by no means a uniform class of materials. In terms of regulation, elastomers have to comply with Article 3, (1a) of Regulation (EC) No 1935/2004 in respect of their health safety when used as FCMs. Rubbers belong to a group of materials, listed in Annex 1 of Regulation (EC) No 1935/2004, for which specific legal measure may be issued [3]. To date, however, there is no harmonised European legislation for rubber; therefore, national laws and regulations apply. The German Federal Institute for Risk Assessment (BfR) has issued Recommendation XXI for commodities based on natural and synthetic rubber, which is acknowledged to be the current state of the scientific and technical knowledge for the conditions under which FCMs made of natural and synthetic rubber meet the requirements stated in Article 3, (1a) of Regulation (EC) No 1935/2004 [4]. Cross-linked TPEs (TPE-V) are only covered by the provisions described above. Non-cross-linked TPEs which have the same composition as plastics are, however, covered by Commission Regulation (EU) No 10/2011, even if their physico-chemical behaviour differs from that of plastics [3,5].

**Figure 1:**
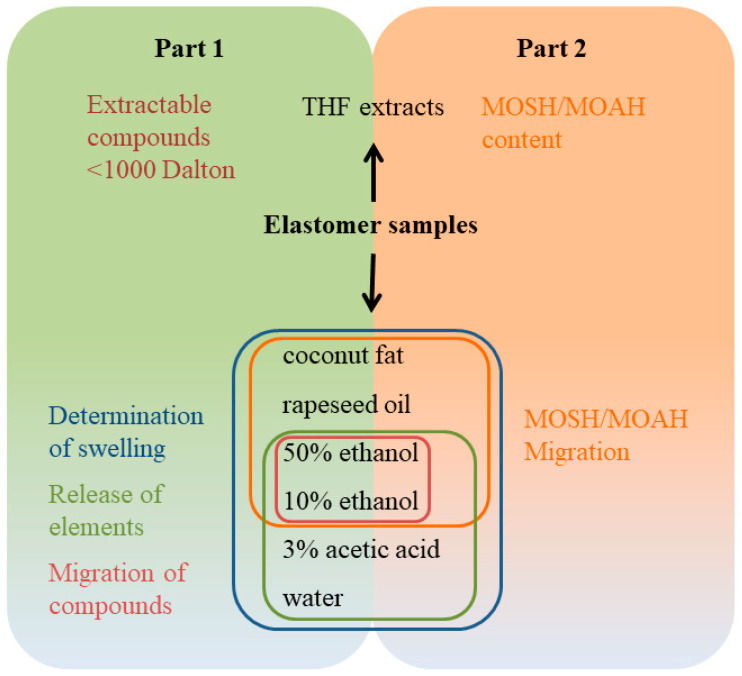
Overview of project—Elastomer samples were extracted (top) and used for migration experiments (bottom). Part 1 (this publication) deals with topics on the left. Part 2 (to be published) deals with the mineral oil hydrocarbons (MOH). MOSH (mineral oil saturated hydrocarbons); MOAH (and mineral oil aromatic hydrocarbons).

Migration of noncovalently bound substances from such chemically diverse polymers as elastomers is dependent on the polarity of the polymer matrix, the substance and the food simulant or extraction solvent. In the past, investigations on the migration of substances from elastomers into food or food simulants has often focussed on selected chemical classes such as primary and secondary aromatic amines [6], (mercapto)benzothiazole [7] and *N*-nitrosamines [8,9,10,11,12]. However, untargeted screening for extractables from FCMs has provided evidence that many more substances may migrate from rubber [13,14,15]. Extractable/migratable substances in rubber may originate from impurities of starting materials or reaction and degradation products. As described above, elastomers represent a very heterogenic class of material. Hence, the variety of compounds, which are to be expected, is vast. For risk assessment, however, the decisive factor is what substances reach the food. To investigate this, food simulants play an important role. It was the aim of the present work to further elucidate which and to what extent substances from different food contact elastomers may find their way into our food. In the first part of the project, ten different elastomer samples were evaluated with respect to the content of extractable compounds, migration of substances into ethanolic food simulants, swelling in food simulants and release of elements in different food simulants. The second part of this work takes a closer look at one part of extractable/migratable substances, namely mineral oil hydrocarbons (MOH). The content of MOH, as well as migration into food simulants, was investigated. Figure 1 illustrates the general content of the study described.

For the quantification of extractable compounds, extracts of elastomer samples were studied. Extraction was optimised to near-completion for all substances under the molecular weight of 1000 Da and as nonexhaustive as possible for larger molecules. Extraction efficiencies were analysed using size exclusion chromatography and evaporative light scattering detection (SEC-ELSD). Extracts were further analysed by comprehensive two-dimensional gas chromatography (GC × GC). This enables the identification or characterisation of components other than mineral oil saturated hydrocarbons (MOSH) and mineral oil aromatic hydrocarbons (MOAH), when coupled to mass spectrometric (MS) detection and estimating the number of components above a limit of interest by using flame ionisation detection (FID). As previously published, Comprehensive GC × GC analysis is a suitable tool to quantify and start identifying extractable and migratable substances from FCMs such as elastomers but also plastics or paper and board [15].

When used as FCMs, the diverse chemical properties of elastomers will have to be taken into account with respect to their respective usage. Swelling of an elastomer in a food simulant is indicative of processes that potentially lead to the enhanced migration of substances from the FCMs into the food. In order to cover the widest possible range of food stuffs, the extent of swelling (measured gravimetrically) was determined in coconut oil, rapeseed oil, 50% ethanol, 10% ethanol, water and 3% acetic acid after 24 h and 10 days of migration time at 40 °C.

The release of elements into the above-mentioned aqueous and ethanolic food simulants was determined by inductively coupled plasma mass spectrometry (ICP-MS).

## 2. Results

### 2.1. Quantification of Extractable Compounds by SEC-ELSD

During optimisation of the extraction method, a selection of samples (EPDM (#2), NR/SBR (#3), TPE (#5)) was extracted with tetrahydrofuran (THF), methyl-tertbutylether (MTBE) and heptane in order to find the ideal solvent for a quantitative extraction. The differences in extraction efficiencies as defined by the amount of substance determined by SEC-ELSD were minor for substances <1000 Da (Table S1). For some of the examples, the amount of MOSH dominates the extracted substances. On the whole, THF was chosen as it seemed more robust for the different samples that were tested. Furthermore, it was assumed THF would be more generic for the polarity range of extractable substances. This could be demonstrated by comparing the extraction efficiencies of all samples for the solvent THF and heptane (Table S2). For the majority of samples, THF extracted a larger amount of substances under 1000 Da.

From a toxicological point of view, substances with a molecular weight >1000 Da are of minor interest as their bioavailability is thought to be negligible [16]. Table 1 shows the extraction efficiency for THF for all samples. The percentage refers to the initial weight of the elastomers. The data show that the extraction is reasonably exhaustive in the first extraction as far as substances <1000 Da are concerned. As there is considerable swelling of many of the samples, the solvent within the swelled elastomer after the first extraction cannot be rinsed off before the second extraction. Therefore, some of the perceived extract in the second extraction may well be due to an exchange of solvent in the swelled elastomer with the fresh solvent. The data in Table 1 also demonstrate that there is considerable variance in the amount of extractable material depending on the sample or type of elastomer. The extractable content with a molecular weight <1000 Da ranged from 1% for the tested PUR (#8) sample to 35 and 45% for the TPE samples (#5 and #6) under investigation.

Data presented in Table 1 also show that the first extraction is far from exhaustive for compounds >1000 Da for certain samples, and subsequent extractions yield more material than the previous one (e.g., NBR #4). This is a desired property for the extraction procedure, as it means there are less interferences with the GC analysis of the relevant low-molecular-weight fraction. In other elastomers, however, the substances of higher molecular weight can also be extracted exhaustively with the chosen conditions (e.g., TPE).

### 2.2. Comprehensive Two-dimensional GC × GC-FID or –MS Analysis of THF Extracts

Extracts were further analysed by comprehensive two-dimensional gas chromatography (GC × GC). This enables the identification or characterisation of components other than MOSH and MOAH when coupled to MS detection and estimating the number of components above a limit of interest by using FID.

**Figure 2:**
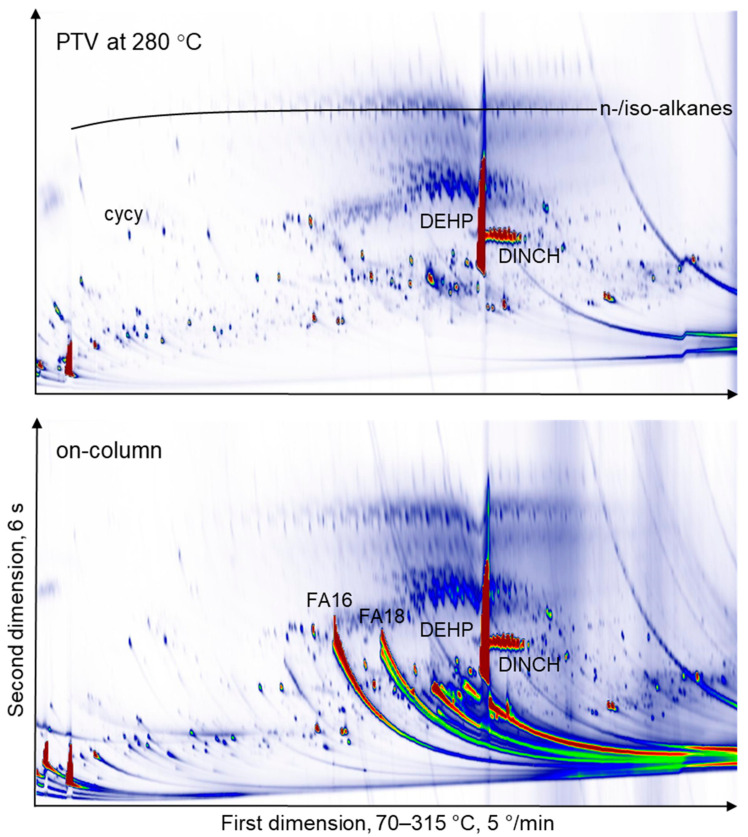
GC × GC-FID (comprehensive two-dimensional gas chromatography coupled with flame ionisation detection) chromatograms of tetrahydrofuran (THF) extract of acrylonitrile butadiene rubber (NBR, #4). The top panel was run with programmable temperature vaporiser (PTV) injection, while for the bottom panel, the extract was injected on-column, leading to more intense peaks of some polar components such as palmitic (FA16) and stearic acid (FA18). A large number of additional peaks are not present in the top panel, which stem from substances formed in the hot PTV injector. cycy: Cyclohexyl cyclohexane, DEHP: Diethylhexyl phthalate, DINCH: 1,2-cyclohexane dicarboxylic acid diisononyl ester, FA16: Palmitic acid, FA18: Stearic acid.

The THF extracts were injected by a programmable temperature vaporiser (PTV), while the MOSH or MOSH and MOAH depleted extracts were injected on-column. PTV injection is useful for the discrimination of low-volatility components and highly polar substances, which are partially adsorbed on glass wool [17,18]. On-column injection, however, mitigates the risk of new substances forming in the hot injector chamber. Due to large signals from, e.g., fatty acids, this can lead to a substantially different appearance of the GC × GC chromatograms, as is shown in Figure 2 of sample NBR (#4). On the bottom panel, the tailing signals from palmitic (FA16) and stearic acid (FA18) are visible very clearly and almost entirely remained in the PTV injector for the top panel. There is no difference between the two panels, for instance, for the large overloaded di-ethylhexyl phthalate (DEHP) and the smaller signal from the 1,2-cyclohexane dicarboxylic acid diisononyl ester (DINCH) isomeric peak pattern. There is no indication of large amounts of signal formation in the chromatogram in the top panel from PTV injection.

**Figure 3:**
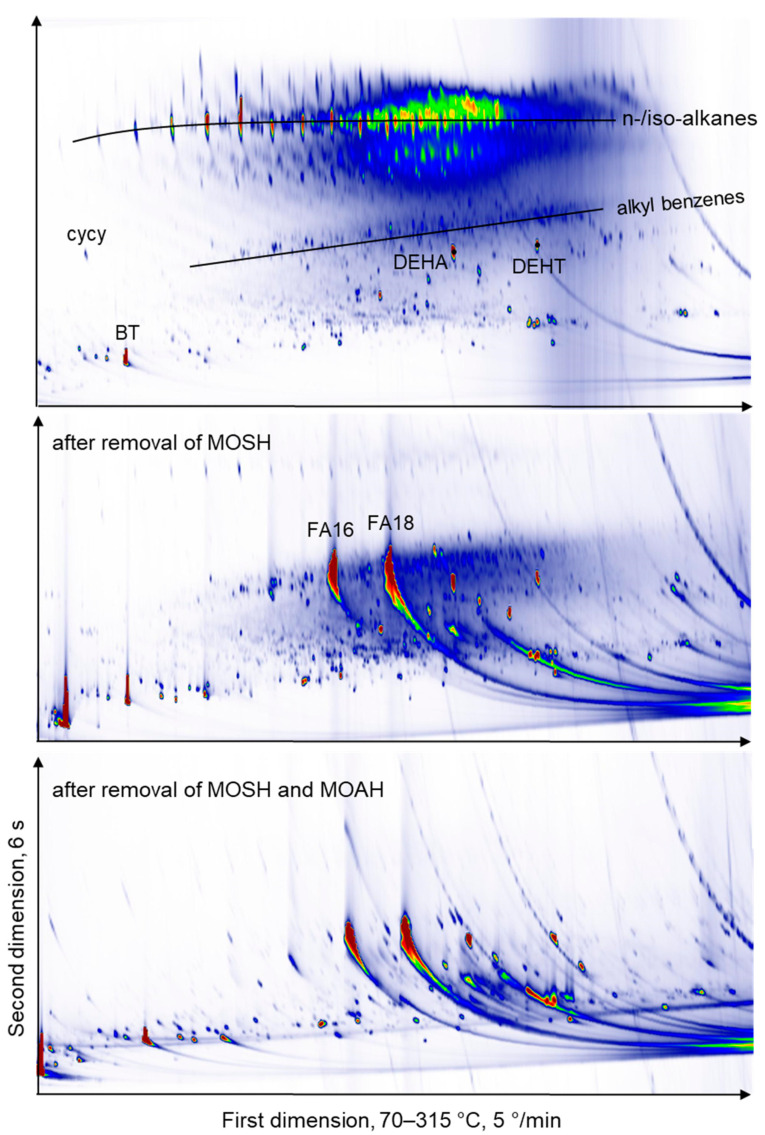
GC × GC-FID chromatogram of a styrene-butadiene-styrene/natural rubber (SBS/NR) sample (espresso maker gasket, #10). The top panel was run with PTV injection explaining the absence of free fatty acids. cycy: Cyclohexyl cyclohexane, BT: Benzothiazole, DEHA: Diethylhexyl adipate, DEHT: Diethylhexyl terephthalate, FA16: Palmitic acid, FA18: Stearic acid.

**Figure 4:**
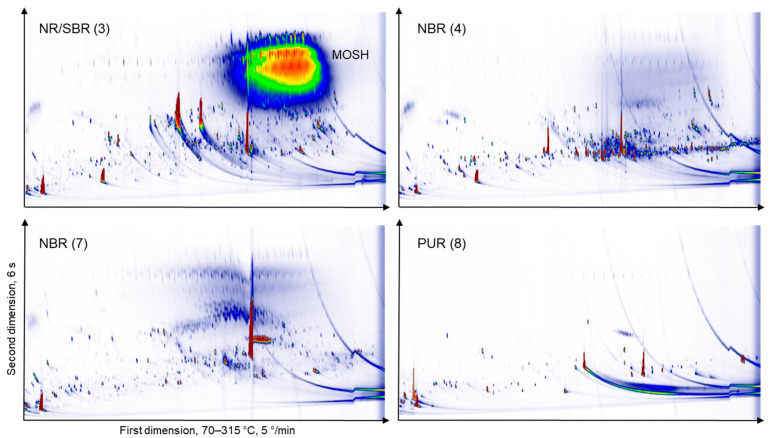
GC × GC-FID chromatograms of a selection of THF extracts without depletion of mineral oil saturated hydrocarbons (MOSH) or mineral oil aromatic hydrocarbons (MOAH) by PTV injection. NR/SBR (natural rubber/styrene-butadiene rubber); NBR (acrylonitrile butadiene rubber); PUR (polyurethane rubber); SBS (styrene-butadiene-styrene).

Samples with a high MOSH content, in addition, were analysed after the removal of MOSH or MOSH and MOAH by liquid chromatography (LC). The main purpose of this approach was to check whether there are other signals co-eluting with MOSH. Figure 3 shows the GC × GC FID chromatograms of the extract of the SBS/NR (styrene-butadiene-styrene/natural rubber) sample (#10, espresso maker gasket), whereby the top panel depicts the raw extract, the middle panel the extract after removal of MOSH and the bottom panel shows the extract after removal of the MOSH and MOAH fractions. The top panel was injected by PTV while the middle and bottom panels were injected on-column. The chromatograms of all samples are shown in Supplementary Figures S1–S11. The data shown in all supplementary figures were collected with PTV injection with the effects described above. In Figure 4, more examples of chromatograms from the THF extracts are shown. There are large differences between the types of elastomers.

The GC × GC-FID chromatograms of the sample extracts were characterised to quantify the substances that were detected. The resulting peak numbers and their content levels can be used for an estimation of the consumer exposure from elastomers, if a reasonable scenario for the contact conditions with the elastomer is applied. The results are summarised in Table 2. The number of substances other than MOSH and MOAH at levels from 1 to 10 ppm and higher than 10 ppm were counted. A few of these signals may be MOAH if they are polyaromatic hydrocarbons with a low degree of alkylation. The MOSH/MOAH content is given as a percentage of the elastomer weight. In addition, the total mass (including MOSH and MOAH) of substances as measured by GC × GC-FID is given as a percentage of the elastomer weight. These values can be compared with the values for compounds <1000 Da as determined by SEC-ELSD (Table 1). The comparison gives an estimate of the coverage GC × GC-FID can achieve relative to the total extractable mass, which ranged between 30 and 80%.

The extracts were all repeated with the use of MS detection. Some spectra of the more intense signals of interest as determined by FID detection were compared to MS databases (NIST and Wiley from 2007) and tentatively identified as far this was possible. Table 2 shows the number of substances that resulted in a mass spectrum of good quality. Roughly half of the mass spectra were from substances without an entry in the libraries. The coverage of substances with tentative assignments ranged between 28 and 61% for all samples. This left between 15 and 132 good-quality spectra with no entry in the databases for all samples.

### 2.3. Substances under 1000 Da in the Ethanol/Water Migrats

**Figure 5:**
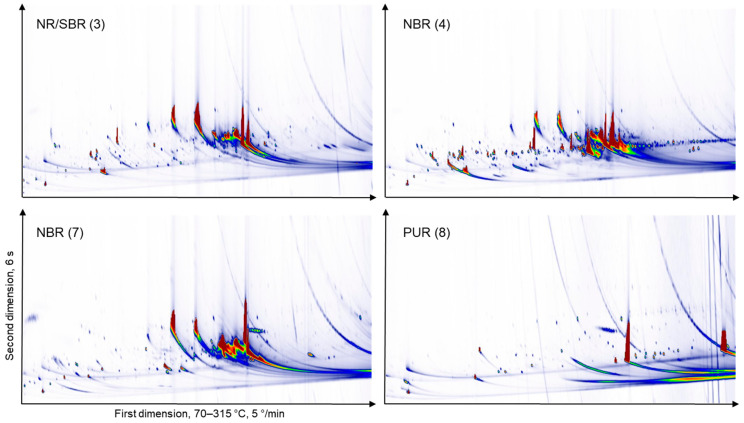
GC × GC-FID chromatograms of a selection of migration solutions in 50% ethanol by on-column injection. The chosen samples correspond to those in Figure 4.

In order to get an idea of how many of the extractable compounds indeed do migrate, a selection of ethanol/water migrats were analysed by SEC-ELSD in order to quantify the content of substances under 1000 Da. Based on the results of the 10 days/50% ethanol migrats, further solutions were tested. The results are shown in Table 3. Most migrats contain a quantifiable amount of substances. The PUR sample (#8) contains a substantial amount of material which migrates. Figure 5 shows the chromatograms of the migration solutions (50% ethanol) with on-column injection of the same four samples as Figure 4. As is to be expected, the more polar substances at the bottom of the contour plots migrate more; MOSH and MOAH are poorly soluble in 50% ethanol. There are a substantial number of signals in the migration solution and there is, again, a large variation between types of elastomers.

### 2.4. Swelling of Elastomer Samples

Swelling of elastomer samples was determined gravimetrically. The percentage weight changes of the samples are listed in Table 4. Where the weight change exceeded 10%, the value is highlighted in grey.

Both 3% acetic acid solution and the vegetable oils had the largest impact on the weight change of the elastomers. Most susceptible to swelling were the tested NR/SBR samples #3 and #9, showing a weight gain of 64 and 41% after 24 h of immersion in 3% acetic acid, respectively. After exposure of these samples to 3% acetic acid for 10 days, on the other hand, sample weight gains were reduced to 31 and 17%, respectively. This observation indicates a decay of the samples over time, possibly through dissolution of fillers.

Both NR and SBR are known to not be oil-resistant and only moderately acid-resistant [19,20]. The lack of swelling resistance towards oils may be attributable to the relative nonpolar character of the polymer. After immersion in rapeseed and coconut oil, the sample weight increased with time. Samples #3 and # 9 (both NR/SBR) showed a swelling of 20 and 12%, respectively, after 24 h of immersion in coconut oil as compared to 64 and 55%, respectively, after 10 days. For the IIR (#1), EPDM (#2) and SBS/NR (#10) samples, a swelling resulting in a more than 10% weight gain was observed only after 10 days of immersion. The weight increase ranged from 11% (IIR, #1) to 24% (SBS/NR (#10). As applicable for all but one sample (#9 after 24 h) that exhibited swelling in oil, coconut oil (mostly C_12_ and C_14_ fatty acids) led to more weight gain than rapeseed oil (mostly C_18_ fatty acids). This observation is consistent with the fact that the penetration rate of organic liquids is reversely correlated to its viscosity [20,21]. Another notable fact is that both TPE samples (#5 and #6) showed a loss of weight after immersion in oil, amounting to 1% for both samples in coconut oil and to 6 and 9%, respectively, in rapeseed oil. This has to be considered in conjunction with the amount of extractable compounds. TPE samples #5 and #6 contain 35 and 45% of extractable compounds with a molecular weight <1000 Da, respectively (see Table 1). It is therefore conceivable that the observed sample weights are the result of oil penetration and extraction of compounds from the polymer at the same time.

The swelling of elastomers in water does not follow the above-described inverse relationship with viscosity. Here, hydrophilic impurities in the polymer affect the water transport. Water-soluble substances are dissolved and form droplets of solution within the polymeric structure, exerting an osmolaric pressure. The influx of water will cease when the existing elastic stresses on the solution droplet reach an equilibrium with the osmolaric pressure [22]. For all investigated samples, the amount of weight increased over, but did in no case exceed, 10%. The lowest amount of swelling was observed in TPE sample #5, amounting to only 0.2%. In conjunction with the observation that a weight loss occurred after oil immersion and that no migrants were detectable in either 10 or 50% ethanol, this indicates that the TPE samples mainly contained hydrophobic impurities/additives. By contrast, NBR sample #7 showed a water absorption of 9.7% after 10 days of immersion. The same sample showed the most pronounced swelling in 10% and 50% ethanol, reaching 11 and 15% after 10 days, respectively. Regulation (EU) No. 10/2011 prescribes for milk 50% ethanol as a food simulant [3]. In discussions over food simulants, manufacturers often argue that 50% ethanol grossly overestimates migration from rubber into milk. In view of the observed swelling as measured by weight gain, this argument cannot be supported. However, Pham et al. [23] showed that ethanol permeates nitrile gloves (NBR) without swelling the material. The authors concluded that for samples from one batch, the diffusion coefficient is strongly correlated to the fractional free volumes in the samples. For samples of different batches, such correlation could not be established, probably due to different engineering processes, different additives, and/or possible sample degradation over time [23]. Further research is needed to investigate to which degree 50% ethanol as a food simulant is suitable to gauge the migration from rubber to milk. It might be necessary to find a more suitable food simulant for milk when testing elastomeric materials.

### 2.5. Release of Elements from Elastomers into Food Simulants

The elastomer samples were immersed for 24 h or 10 days at 40 °C according to category 2 (food contact up to 24 h) and category 1 (food contact longer than 24 h) of BfR Recommendation XXI [4], respectively, and the release of 20 elements was determined as described in materials and methods. Tested food simulants were water, 3% acetic acid, 10% ethanol and 50% ethanol. The highest migration of elements was detected in 3% acetic acid and 50% ethanol. The complete results (mean of two migrations per sample) are shown in Table S3. Values for lead (Pb), aluminium (Al), zinc (Zn), nickel (Ni), manganese (Mn) and arsenic (As) were detected at levels above those stated either in Commission Regulation (EU) No 10/2011 for FCMs made of plastic [3] or the EDQM guidelines for metals and alloys used in food contact materials and articles [24] (see Table 5).

Al can cause neurotoxic and nephrotoxic effects and damages the developing embryonic nervous system [25]. Based on the fact that Al accumulates in the human body (mainly in bone tissue) and the above-described negative effects on humans, the European Food Safety Authority (EFSA) has derived a weekly tolerable intake (TWI) of 1 mg/kg bodyweight (BW) [26]. Under application of an allocation factor of 10%, this value was used to derive a specific migration limit (SML) of 1 mg/kg food (simulant) for FCMs made of plastics [3]. In rubber, aluminium salts may be present as fillers or may be used as precipitating agents. The release limits for Al were only exceeded in the migrats of two samples after 10 days migration in 3% acetic acid (sample #4 (NBR) and #10 (SBS/NR) amounting to 1.3 and 1.2 mg/L, respectively (see Table 6)). These are no major exceedances of the limit of 1 mg/kg food or food simulant stated in Regulation (EU) No 10/2011. Furthermore, the true surface–volume-ratio has not been established. For sample #4, a pre-product, the final use of the elastomer is not known. For sample #10, an espresso maker gasket, the test time would be 24 h rather than 10 days [4]. Consequently, no imminent health risk due to the release of Al is to be expected from these samples.

Due to its toxicity to reproduction and development, EFSA has derived a tolerable daily intake (TDI) of Ni of 13 μg/kg BW [27]. The 95th percentile upper bound acute exposure to Ni through food consumption ranges from 5.4 in the very elderly to 40.8 μg/kg BW per day in toddlers. Considering that for some age groups, the TDI is already exceeded by food consumption, any further intake should be as low as possible. On the basis of a previously established TDI of 2.8 μg/kg BW [28] and under the application of an allocation factor of 10%, a specific release limit of 20 μg/kg food (simulant) was incorporated in Regulation (EU) No 10/2011 [29]. Whether a new specific release limit will be put in place remains to be seen. Two of the samples investigated here showed the migration of Ni exceeding 20 μg/L, namely sample #7 (NBR) and sample #9 (NR/SBR; preserving jar gasket). With 114 μg/L, the NBR sample showed an especially high migration after 10 days migration time in 3% acetic acid, but also, immersion in 50% ethanol produced a Ni concentration more than 100% above the current limit (47 μg/L). Ni may have been intentionally added to the rubber by using a nickel-containing stabiliser such as nickel dibutyldithiocarbamate [30].

The same sample (NBR, #7) was the only one that showed an elevated release of manganese (Mn) in 3% acetic acid (see Table 6). Mn is one of the metals known as “rubber toxin” as it accelerates the fission of the macromolecules in natural rubber [19]. Its presence in the rubber is most likely unintentional. For humans, Mn is an essential mineral, which is involved in numerous metabolic pathways. EFSA has proposed an adequate daily intake of 3 mg/day for adults [31]. The average dietary exposure amounts to 2.8 mg for a 70 kg person [32]. Intoxications with manganese cause symptoms similar to those of Parkinson’s disease. Whilst the highest exposures to manganese occur in occupational settings, evidence is growing that exposures through environmental and nutritional sources, drinking water or airborne contaminations may also be the cause of toxicities [33]. An acute intoxication due to Mn from the investigated material is, however, unlikely.

Zn-containing accelerators are frequently used in the manufacturing of rubber [34]. It is hence not surprising that Zn is the most abundant element detected in the migration solution of rubber (see Table 6), whereby 3% acetic acid as a food simulant produces the highest values. After 10 days of immersion, values range from 0.1 (PUR, sample #8) to 333.0 mg/L (NBR, sample #4). Zn is an essential element for human life. It constitutes catalytic and/or structural functions in all six enzyme classes. Furthermore, it is involved in intracellular signalling [35]. Whilst severe deficiency is detrimental, chronic high intake can lead to the manifestation of neurological disorders [36]. Regulation (EU) No 10/2011 prescribes a limit of a maximum of 5 mg/kg food or food simulant applying an allocation factor of 20% to the tolerable upper level of zinc of 25 mg/day for adults [3,37,38]. Although for 3% acetic acid solution and 50% ethanol, values exceeding the set limit of 5 mg/kg food simulant are frequent, this was true for only one sample after 10 days immersion in water (NR/SBR, sample #9, 6.7 mg/L, see Supplementary Table S3) and for none of the samples immersed in 10% ethanol. Migration of Zn from rubber FCMs is often unpreventable. The migration conditions selected here represent a worst-case scenario, which in most cases will overestimate the real migration. Nevertheless, the possible migration of Zn must be borne in mind especially when the FCMs in question are to be put in contact with acidic foods.

Arsenic is carcinogenic to humans [39]. Due to its genotoxic and carcinogenic properties, it is not possible to determine a safe dose. Exposure to As at low levels via food and drinking water does occur inevitably. According to consumption data, compiled by EFSA, the intake of inorganic As amounts from 0.11 up to 0.38 μg per kg BW per day [40]. EFSA has derived a benchmark lower dose level (BMDL_01_) of 0.3 to 8 μg/kg BW per day for lung, skin, and bladder cancer and skin lesions. Therefore, the possibility of an elevated risk for some consumers cannot be excluded [41]. The European Council recommends, based on the BMDL_01_ of 0.3 μg/kg BW, a maximum release limit of 20 μg As/kg food, assuming the BW to be 60 kg, a daily consumption of 1 kg and an allocation factor of 10% [24]. Due to the mutagenic and carcinogenic properties of inorganic As, BfR recommends that the release from enamelled barbeque grills should not be detectable [42]. A limit of quantification in the range of 1 μg/L should be achievable for most laboratories. Two out of the nine tested samples (#7, NBR and #10 SBS/NR) showed levels above 1 μg/L following 10 days immersion in 3% acetic acid (see Table 6).

Pb is a heavy metal which is, according to the current knowledge, not directly genotoxic [43]. However, the International Agency for Research on Cancer (IARC) has classed Pb as “possibly carcinogenic to humans” (class 2B) and inorganic Pb compounds as “probably carcinogenic to human” (class 2A) [44]. Furthermore, Pb has been classified as toxic to reproduction Class 1A (“may damage fertility, may damage the unborn child“) according to Regulation (EC) 1272/2008 (CLP Regulation) [45]. As no safe intake level regarding the developmental neurotoxicity in children can be derived, the ALARA (as low as reasonably achievable) principle should be used when limits are set for Pb release from FCMs [46]. The limit of 10 μg/L as proposed by the European Council [24] is in agreement with the “discussion starting value” for ceramics proposed by the European Reference Laboratory for FCMs [47]. This value was exceeded by six out of the nine tested samples (see Table 6). Sample #3 (NR/SBR) and #10 (SBS/NR) were found to exceed migration values of 500 μg/L after 10 days of immersion in 3% acetic acid and 50% ethanol, respectively.

In summary, the release of certain elements (e.g., Pb, Ni, Zn and As) from FCMs made of rubber bears the potential of an exposure risk. This notion has also been shared by researchers investigating pharmaceutical-grade rubber stoppers [48].

## 3. Materials and Methods

### 3.1. Materials

#### 3.1.1. Solvents

Acetone (LC grade), ethanol (LC grade) and methanol (LC-MS grade) were purchased from Merck (Darmstadt, Germany). Acetic acid was obtained from Carl Roth GmbH & Co. KG (Karlsruhe, Germany). Hexane CHROMASOLV (LC grade), heptane puriss, cyclohexyl cyclohexane, eicosane methyl ester, biphenyl and 1,3-diphenoxy benzene. p.a. were purchased from Sigma-Aldrich (Taufkirchen, Germany). HPLC-grade water (18.2 MΩ∙cm output quality) was obtained from Milli-Q water purification equipment (Merck Millipore, Darmstadt, Germany). THF for synthesis (from EGT Chemie AG, Tägerig, Switzerland) was distilled prior to use in order to remove the antioxidant 3,5-di-tert-butyl-4-hydroxytoluene (BHT). MTBE for synthesis was obtained from Brenntag Schweizerhall AG (Basel, Switzerland) and distilled in-house. Dichloromethane was from J.T. Baker (Deventer, The Netherlands).

#### 3.1.2. Elastomer Samples

Semi-finished products: IIR sheet, EPDM sheet, PUR sheet, NR/SBR food sheet, NBR sheet, rectangular thermoplastic elastomer profile (TPE, SEBS).

Finished elastomer products (preserving jar sealing ring (red, NR/SBR), bottle swing stopper sealing ring (green, TPE, SEBS), espresso maker sealing ring (SBS/NR)) were purchased from retail sale. Semi-finished products were obtained from Reichelt Chemietechnik GmbH + Co., Heidelberg, Germany and Dichtungstechnik Bremen, Germany. It is unknown to which finished products the semi-finished products were processed. The samples were chosen to give an overall view on elastomers and not to make statements on individual products.

### 3.2. Methods

#### 3.2.1. Extraction with THF

A sample of 500 mg cut into pieces of approximately 4 × 4 mm was extracted with 5 mL THF for 4 days in a water bath at 55 °C. For the second extraction, the THF was removed and transferred into a dark glass bottle. The remaining sample was rinsed with THF and then extracted again with 5 mL THF for 7 days at 55 °C. These steps were repeated for a third extraction.

#### 3.2.2. Migration Testing According to BfR Recommendation XXI

Migration testing was carried out according to BfR Recommendation XXI for FCM-based natural and synthetic rubber [4]. Surface areas of elastomer samples were determined including cut edges. Migration experiments were carried out by total immersion, whereby a ratio of 2 mL simulant per cm^2^ surface was applied. Corresponding to categories 1 (food contact time >24 h) and 2 (food contact time ≤24 h), samples were subjected to migration at 40 °C for 10 days and 24 h, respectively. Used food simulants were deionised water, 3% acetic acid, 10% ethanol in water, 50% ethanol in water, coconut oil and rapeseed oil. Further details regarding the migration procedure are described by Kühne et al. [12].

For the determination of elements in food simulants, samples were placed in polypropylene tubes rather than glassware in order to avoid contamination. Samples were prepared in duplicates.

#### 3.2.3. SEC-ELSD

A volume of 20 μL of the extracts were injected into a SEC-ELSD (Phenogel 5 μm, 500 Å, 300 × 7.8 mm separation column, Phenogel 5 μm, linear mixture, 50 × 7.8 mm pre-column, Phenomenex, Torrance, USA; Accela HPLC system, Thermo Scientific, Milano, Italy; ELSD 90, VWR International, Dietikon, Switzerland). A flow rate of 800 μL/min was applied at 40 °C column temperature. The ELSD gain was set at 2, the air pressure at the nebuliser was 3 bar and the temperature of the drift tube hold at 40 °C. For quantification, an external calibration was used with di-2-ethylhexyl adipate. The calibration curve was exponential. The section of the 1000 Da cut was determined with an epoxy resin as has been described previously [49]. The cut contains an uncertainty of approximately 350 Da (determined by different polyadipates), as SEC separates on molecular volume rather than molecular mass.

#### 3.2.4. Determination of Swelling Caused by Food Simulants

Swelling of the elastomer samples was determined gravimetrically. Samples were weighed before the migrating and after 1 and 10 days, respectively. After migration, samples were carefully dried with tissue.

#### 3.2.5. GC × GC-FID/GC × GC-MS

For the extraction, 500 mg of sample material was extracted with 5 mL of THF and 100 μL of internal standard solution containing 50 mg/L cyclohexyl cyclohexane, 50 mg/L eicosane methyl ester, 5 mg/L biphenyl and 5 mg/L 1,3-diphenoxy benzene. The internal standards are therefore at a concentration of 1 and 10 mg/kg sample. The response of these internal standards allows for a semi-quantitative determination of all other substances in the FID chromatograms. A volume of 0.6 μL was injected into the programmable temperature vaporiser (PTV) injector equipped with a liner packed with glass wool at an initial temperature of 60 °C. The injector temperature was increased to 280 °C during the splitless transfer of 1 min. In order to verify there was no substantial formation of decomposition products in the hot PTV injector or discrimination against high boiling substances, 0.6 μL of the extract was also injected on-column. For the analysis of the migration solutions, 2 mL 50% ethanol simulant was extracted with 2 mL MTBE prior to injection of the organic phase.

For selected samples (see Figures S1–S11), 10 μL of the extracts were injected into LC as was done for the MOSH/MOAH analysis. After the elution of MOSH or the MOSH and MOAH fraction (see Figure S11), the column flow was reversed and the eluent switched to 100% dichloromethane. A backflush fraction of 1100 μL was collected and re-concentrated under a nitrogen flow to 200 μL. The extract of the TPE swing stopper (sample #6) was an exception as it was viscous. This extract was diluted in hexane 1:10 and 90 μL was injected into LC. A volume of 8 μL was injected on-column onto the GC × GC system.

GC and MS conditions were selected as previously described [50]. The GC × GC instrument consisted of a TRACE GC from Thermo Scientific (Milan, Italy) and a two-stage thermal loop modulator ZX2 from Zoex Corporation (Huston, TX, USA). Detection was performed either with FID or the Bench TOF-dx mass spectrometer (Markes International, Llantrisant, UK). An uncoated deactivated pre-column of 1.5 m × 0.53 mm. was connected to a 15 m × 0.15 mm i.d. DB-17 first-dimension separation column (50% phenyl methyl polysiloxane of 0.15 μm film thickness; Agilent, Santa Clara, CA, USA) followed by a 3.2 m × 0.15 mm i.d. apolar second-dimension separation column coated in-house with a 0.055 μm film of PS-255 (Sigma-Aldrich, Taufkirchen, Germany; discontinued article). The modulator involved a constant stream of 7.5–10 L/min air cooled to −84 °C and hot air at 370 °C pulsed every 6 s during 350 ms on the inlet of the second-dimension column of which the first 1 m was used as the modulator delay loop.

#### 3.2.6. Determination of Elements by ICP-MS

The release of 20 elements (Li, Be, Al, V, Cr, Mn, Fe, Co, Ni, Cu, Zn, As, Mo, Cd, Sn, Sb, Ba, Hg, Tl and Pb) was determined by ICP-MS. Tested food simulants were water, 3% acetic acid, 10% ethanol and 50% ethanol.

After release testing, simulants were spiked with 150 μL of internal standard solution (ruthenium ions, 5 mg/L) and were diluted 1:10 with 3.5% nitric acid (containing 200 μg/L gold ions). Simulants containing alcohol were sonicated at 70 °C for 90 min to evaporate ethanol. The volume of the solution was readjusted with 3.5% nitric acid.

Elements up to a weight of 117 u were measured with the collision cell technology as kinetic energy discrimination (KED) and a mixed gas of helium with 2% hydrogen on an ICP-MS system (Thermo Fisher Scientific, iCapQ and X-Series II (PlasmaLab 2.5.11.321)). In order to enhance the sensitivity of various elements at X-Series II, the Pole Bias was switched between 0 or 3 V when KED-Modus was used. Elements heavier than 117 u were measured in standard mode. The system was operated with 1550 W of RF-power. The gas flow of the nebuliser, auxiliary gas, and cooling gas were set to 1.1, 0.7 and 14 L/min, respectively. Samples were measured with a dwell time of 0.01 s with 100 sweeps per reading. To account for dilution inaccuracies during sample preparation, the internal ruthenium standard was used to calculate a correction factor. A mixed internal standard solution containing rhodium and bismuth in 10% isopropanol and 3.5% nitrous acid was used as the injection standard with a concentration of 5 μg/L. The latter was added immediately before the solution was nebulised via the autosampler system (Elemental Service & Instruments GmbH, prepFAST). Limits of quantification (LOQ, see Table S4) were determined using the blank value method. Data were processed with the Qtegra software (Thermo Fisher Scientific, Version 2.8.2944.202 64 Bit) and Excel (Microsoft).

## 4. Conclusions

The work presented here demonstrates that a potentially high number of substances may migrate from rubber FCMs into food. For food-related risk assessment, the use of food simulants plays a vital role: On the one hand, to standardise testing conditions, and on the other hand, for the ease of analysis without food matrix. Despite all simplification, it must, however, be guaranteed that the testing conditions do reflect a worst-case scenario. It was shown that food simulants harbour the potential to morphologically alter or even disintegrate elastomeric materials. If food, which is intended to be brought into contact with the respective material, causes such alterations, this needs to be taken into account when the safety of the FCMs is assessed. If necessary, the material needs to be improved or substituted in order to be fit for purpose.

Elastomer food contact materials can be the source of food contamination with aluminium and heavy metals. Especially, contamination with lead was frequently detected in the samples investigated in this study. In contrast to other FCMs, there are no legal limits for elements released from elastomers in European legislation. It is therefore important that national regulations address this issue to keep consumer exposure as low as possible. The current BfR recommendation XXI on commodities based on natural and synthetic rubber [4] provides a limit for zinc and lead in the finished material. A revised version will be published shortly. Therein, migration values for Al (1 mg/kg food), Pb (0.01 mg/kg food) and Zn (25 mg/kg food) will be published. Typical formulations of rubbers do not usually contain more than 20 ingredients. The number of extractable compounds, however, is by far larger and very variable across the investigated elastomers. Vulcanisation, production processes or aging may lead to degradation and reaction products. The risk assessment of migrating substances from rubber FCMs therefore needs to consider these nonintentionally added substances (NIAS) alongside the intentionally added substances. Given the high number of potentially migrating NIAS, concepts for risk assessment of elastomeric materials may need to be adapted.

## Figures and Tables

**Table 1:** Extraction in percent of elastomer weight for three consecutive extractions.

Sample Type	Elastomer (Sample #)	Labelled as FCM	Extractable Content with THF [%]					
			<1000 Da			>1000 Da		
			Extraction #			Extraction #		
			1	2	3	1	2	3
pre-product	IIR (1)	no	10	1.5	0.4	2.4	1.1	1.4
pre-product	EPDM (2)	yes	28	3.3	0.2	3.0	0.4	0.3
pre-product	NR/SBR (3)	yes	6	1.8	0.4	2.5	3.0	4.5
pre-product	NBR (4)	yes	6.5	1.2	0.3	2.6	5.2	7.3
pre-product	TPE (5)	yes	35	2.5	0.1	30	2.6	0.2
gasket, bottle swing stopper	TPE (6)	yes	45	4.3	0.3	28	2.0	0.3
pre-product	NBR (7)	yes	5.5	1.2	0.2	2.5	0.5	0.2
pre-product	PUR (8)	yes	1.0	0.2	0.0	1.6	0.7	0.2
gasket, preserving jar	NR/SBR (9)	yes	7.5	2.5	0.9	8.0	13	7.4
gasket, espresso maker	SBS/NR (10)	yes	5.0	0.7	0.2	0.8	1.8	2.2

THF (tetrahydrofuran); FCM (food contacts material); IIR (isobutylene-isoprene rubber); EPDM (ethylene propylene diene monomer rubber); NR/SBR (natural rubber/styrene-butadiene rubber); NBR (acrylonitrile butadiene rubber); TPE (thermoplastic elastomer); PUR (polyurethane rubber); SBS (styrene-butadiene-styrene).

**Table 2:** Number of substances found in the GC × GC-FID chromatograms and number of spectra obtained by GC × GC-MS.

Sample (#)	Number of Substances (excl. MOSH/MOAH)		% of Elastomer Weight (signals incl. MOSH/MOAH)	Number of Mass Spectra	% Tentatively Assigned Spectra
	1–10 ppm	>10 ppm	[%]		[%]
IIR (1)	280	53	6.4	80	61
EPDM (2)	147	7	12.0	56	59
NR/SBR (3)	327	78	3.1	97	44
NBR (4)	471	194	2.1	186	29
TPE (5)	57	4	19.3	40	45
TPE (SEBS) (6)	103	22	14.0	48	48
NBR (7)	484	68	2.6	158	47
PUR (8)	54	17	0.8	42	64
NR/SBR (9)	569	121	4.2	101	28
SBS/NR (10)	487	154	4.1	83	42

Coverage by GC × GC-FID of total extractable mass can be seen by comparison to the extraction of substances <1000 Da in Table 1. SEBS (styrene-ethylene-butadiene-styrene)

**Table 3:** Content of substances under 1000 Da in ethanol/water migrats determined by size exclusion chromatography-evaporative light scattering detection (SEC-ELSD).

Elastomer (sample #)	SEC * < 1000 Da	10% Ethanol		50% Ethanol		10% Ethanol		50% Ethanol	
		1 day	10 days	1 day	10 days	1 day	10 days	1 day	10 days
	[mg/dm^2^]	[mg/dm^2^]				mg migrated [%] **			
IIR (1)	900	-	-	-	12	-	-	-	1.3
EPDM (2)	2581	-	-	-	14	-	-	-	0.5
NR/SBR (3)	627	-	6.5	18	62	-	1.0	2.9	9.9
NBR (4)	718	-	7.5	27	95	-	1.0	3.8	13.3
TPE-S (5)	3169	-	-	-	<6	-	-	-	<0.2
TPE-S (6)	3938	-	-	-	<6	-	-	-	<0.2
NBR (7)	713	-	7.0	20	76	-	1.0	2.8	10.7
PUR (8)	92	11	24	33	75	12	26	36	81
NR/SBR (9)	982	-	-	-	20	-	-	-	2.0
SBS/NR (10)	651	-	-	-	22	-	-	-	3.3

* THF extract; ** related to THF extract <1000 Da; dashes indicate that no measurement was done, as it was considered irrelevant due to the measurement of 50% ethanol after 10 days.

**Table 4:** Swelling of elastomer samples in food simulants in % as determined by weight gain after 24 h and 10 days of migration time. Weight changes exceeding 10% are highlighted in grey.

Elastomer Type (Sample #)	H_2_O		3% Acetic Acid		10% Ethanol		50% Ethanol		Coconut Oil		Rapeseed Oil	
	24 h	10 d	24 h	10 d	24 h	10 d	24 h	10 d	24 h	10 d	24 h	10 d
IIR (**1**)	0.5	1.9	1.2	5.4	0.5	2.1	0.8	2.4	5.1	16.4	2.1	6.7
EPDM (**2**)	0.2	0.6	1.9	5.8	0.2	0.7	0.2	0.5	2.6	10.7	−2.4	0.9
NR/SBR (**3**)	0.5	1.4	64.4	31.0	0.6	1.5	0.7	−1.3	20.0	64.2	17.2	54.2
NBR (**4**)	1.2	3.7	3.8	3.8	1.8	4.8	3.3	4.8	2.8	9.1	2.0	6.8
TPE-S (**5**)	-	-	-	-	-	-	-	-	−0.9	−0.9	−5.9	−8.9
TPE-S (**6**)	0.1	0.2	2.0	17.4	0.1	0.3	0.3	0.4	−2.7	−2.4	−5.9	−4.5
NBR (**7**)	2.3	9.3	6.6	11.5	3.7	11.4	6.8	15.3	1.5	4.4	1.0	3.2
PUR (**8**)	1.6	2.7	2.2	2.0	2.5	2.8	15.8	15.3	0.6	−0.6	−0.3	−0.8
NR/SBR (**9**)	0.5	1.4	40.9	16.8	0.6	1.5	3.9	1.9	11.7	55.4	16.8	49.3
SBS/NR (**10**)	0.4	1.5	1.9	4.2	0.8	1.8	1.2	2.9	5.2	24.1	4.6	19.2

**Table 5:** Maximal permitted release limits for elements as stated in Regulation EU No 10/2011 and the Council of Europe guideline for metals and alloys used in food contact materials and articles.

Element	Maximal Permitted Migration	Source/Comments
Al	1000 μg/kg food (simulant)	Specific migration limit taking into account other sources of intake (EU Regulation No 10/2011)
Ni	20 μg/kg food (simulant)	Specific migration limit taking into account other sources of intake (EU Regulation No 10/2011); (Regulation EU 2017/752)
Zn	5000 μg/kg mg/kg food (simulant)	Specific migration limit taking into account other sources of intake (EU Regulation No 10/2011)
Mn	600 μg/kg food (simulant)	Specific migration limit taking into account other sources of intake (EU Regulation No 10/2011)
As	2 μg/kg food (simulant)	Release limit for As from metals and alloys
Pb	10 μg/kg food (simulant)	Release limit for Pb from metals and alloys

**Table 6:** Release of selected elements from elastomer samples after 24 h and 10 days of migration time in 3% acetic acid and 50% ethanol.

Sample Type (sample #)	Food Simulant	Migration Time	Unit	Al	Mn	Ni	Zn	As	Pb
IIR (**1**)	3% ac. acid	24 h	[μg/L]	50.27	<LOQ	2.94	1351	0.01	0.61
		10 d	[μg/L]	20.96	0.70	2.06	3115	<LOQ	1.30
	50% ethanol	24 h	[μg/L]	7.00	0.25	0.71	217	<LOQ	2.56
		10 d	[μg/L]	19.85	0.98	3.37	553	<LOQ	3.61
EPDM (**2**)	3% ac. acid	24 h	[μg/L]	63.62	<LOQ	1.05	2401	0.01	0.38
		10 d	[μg/L]	137.93	0.97	11.86	2837	<LOQ	0.34
	50% ethanol	24 h	[μg/L]	3.74	<LOQ	0.93	410	<LOQ	<LOQ
		10 d	[μg/L]	7.75	1.15	0.46	919	<LOQ	3.03
NR/SBR (**3**)	3% ac. acid	24 h	[μg/L]	47.59	0.29	2.89	4882	0.02	149.27
		10 d	[μg/L]	386.99	5.16	10.86	22,304	0.12	558.99
	50% ethanol	24 h	[μg/L]	<LOQ	1.86	3.47	2779	<LOQ	108.70
		10 d	[μg/L]	<LOQ	3.58	6.10	4693	<LOQ	89.47
NBR (**4**)	3% ac. acid	24 h	[μg/L]	288.75	20.35	2.46	137,029	0.12	38.84
		10 d	[μg/L]	1265.49	58.68	8.61	333,017	0.36	54.73
	50% ethanol	24 h	[μg/L]	<LOQ	0.40	0.69	2563	<LOQ	30.07
		10 d	[μg/L]	<LOQ	1.88	7.75	9885	<LOQ	57.89
**TPE-S (5)**					No sample available				
TPE-S (**6**)	3% ac. acid	24 h	[μg/L]	13.67	0.48	4.07	95	0.01	0.33
		10 d	[μg/L]	15.09	2.26	2.78	342	<LOQ	0.23
	50% ethanol	24 h	[μg/L]	<LOQ	<LOQ	2.35	133	<LOQ	<LOQ
		10 d	[μg/L]	4.17	0.92	2.04	1433	<LOQ	40.11
NBR (**7**)	3% ac. acid	24 h	[μg/L]	224.70	264.11	18.41	143,810	0.52	8.26
		10 d	[μg/L]	848.44	1068.35	114.42	291,942	1.70	29.09
	50% ethanol	24 h	[μg/L]	<LOQ	<LOQ	20.95	1062	<LOQ	8.62
		10 d	[μg/L]	<LOQ	1.03	47.21	2469	<LOQ	14.21
PUR (**8**)	3% ac. acid	24 h	[μg/L]	6.85	<LOQ	0.41	<LOQ	0.004	<LOQ
		10 d	[μg/L]	5.35	<LOQ	<LOQ	106	<LOQ	0.03
	50% ethanol	24 h	[μg/L]	<LOQ	<LOQ	<LOQ	129	<LOQ	<LOQ
		10 d	[μg/L]	10.76	0.70	12.53	412	<LOQ	11.38
NR/SBR (**9**)	3% ac. acid	24 h	[μg/L]	221.09	1.30	0.47	44,568	0.09	<LOQ
		10 d	[μg/L]	290.37	2.97	<LOQ	108,417	0.28	0.08
	50% ethanol	24 h	[μg/L]	<LOQ	1.60	<LOQ	3626	<LOQ	0.17
		10 d	[μg/L]	39.07	7.87	30.68	9621	<LOQ	3.82
SBS/NR (**10**)	3% ac. acid	24 h	[μg/L]	288.65	46.37	5.32	91,447	0.79	40.02
		10 d	[μg/L]	1234.87	219.53	18.86	242,867	3.62	204.29
	50% ethanol	24 h	[μg/L]	<LOQ	<LOQ	11.92	3651	0.22	232.86
		10 d	[μg/L]	15.51	5.32	19.71	5575	0.47	540.52

LOQ (limit of quantification).

## Data Availability

Data is contained within the article or supplementary material.

## Supplementary Materials

The following are available online: Figures S1–11: GC × GC-FID chromatograms of the extracts from all samples, Table S1: Comparison of extraction efficiency of tetrahydrofuran (THF), heptane (C7), and methyl-tertbutylether (MTBE) as determined by LC-ELSD, Table S2: Comparison of extraction between THF and heptane for all samples, Table S3: Levels of elements in food simulants, Table S4: Limits of quantification (LOQ) in μg/L for elements in different matrices as determined by blank value method.
