# Supplementary material for: Characterisation of Elastomers as Food Contact Materials–Part 1: Quantification of Extractable Compounds, Swelling of Elastomers in Food Simulants and Release of Elements
Source: Molecules. 2021 Jan 19;26(2):509. doi: 10.3390/molecules26020509 (PMC7835956; doi:10.3390/molecules26020509)
Supplement: Supplementary file 1 [file molecules-26-00509-s001.pdf]

Table S1. Comparison of extraction efficiency of tetrahydrofuran (THF), heptane (C7), methyl-tertbutylether (MTBE) as determined by LC-ELSD.

Sample (sample #)	Extraction solvents	Extractable mass [%]					
		<1000 Da			>1000 Da		
		extraction #			*extraction #*		
		1	*2*	*3*	*1*	*2*	*3*
EPDM (2)	THF/THF/THF	28	3.3	0.2	5.0	0.4	0.3
	C7/C7/THF	29	3.2	0.4	3.2	0.3	0.1
	MTBE/MTBE/THF	29	3.8	0.4	4.3	0.4	0.1
NR/SBR (3)	THF/THF/THF	6.0	1.8	0.4	2.5	3.0	4.5
	C7/C7/THF	5.4	0.9	0.6	4.3	4.6	6.7
	MTBE/MTBE/THF	6.5	1.4	0.4	2.5	1.0	4.4
TPE (5)	THF/THF/THF	35	2.5	0.1	30	2.6	0.2
	C7/C7/THF	32	7.0	1.3	8.0	4.0	10
	MTBE/MTBE/THF	32	3.3	0.3	23	3.0	7.0

MTBE was extracted for 4 days for the first time and 7 days for the second at 55 °C and heptane at 80 °C. The third and last extraction was always done with THF for 7 days at 55 °C.

Table S2. Comparison of extraction between THF and heptane for all samples.

sample (sample #)	Extractable mass [%]			
	<1000 Da		>1000 Da	
	**THF**	**heptane**	**THF**	**heptane**
IIR (1)	10	9.0	2.4	3.5
EPDM (2)	28	29	3.0	3.2
NR/SBR (3)	6.0	5.4	2.5	4.3
NBR (4)	6.5	4.0	2.6	2.5
TPE (5)	35	32	30	8.0
TPE (SEBS) (6)	45	49	28	1.0
NBR (7)	5.5	4.5	2.5	0.05
PUR (8)	1.0	0.1	1.6	0.1
NR/SBR(9)	7.5	4.5	8.0	11
SBS/NR (10)	5.0	3.6	0.8	1.0

Table S3. Levels of Elements in food simulants

Sample type (sample #)	Food simulant	Migration time	unit	Li	Be	Al	V	Cr	Mn	Fe	Co	Ni	Cu	Zn	As	Mo	Cd	Sn	Sb	Ba	Hg	Tl	Pb
IIR (1)	AcAc	24h	[μg/L]	<LOQ	<LOQ	50.27	0.10	<LOQ	<LOQ	11.70	<LOQ	2.94	<LOQ	1351	0.01	0.04	0.06	<LOQ	0.03	0.35	<LOQ	0.001	0.61
	AcAc	10d	[μg/L]	<LOQ	<LOQ	20.96	0.22	<LOQ	0.70	20.10	<LOQ	2.06	2.88	3115	<LOQ	<LOQ	0.09	<LOQ	0.07	1.32	<LOQ	0.001	1.30
	EtOH 10%	24h	[μg/L]	<LOQ	<LOQ	<LOQ	<LOQ	<LOQ	<LOQ	<LOQ	<LOQ	<LOQ	<LOQ	211	<LOQ	<LOQ	0.02	<LOQ	<LOQ	<LOQ	<LOQ	0.006	1.89
	EtOH 10%	10d	[μg/L]	<LOQ	<LOQ	21.32	<LOQ	<LOQ	<LOQ	<LOQ	<LOQ	1.37	<LOQ	562	<LOQ	<LOQ	0.20	<LOQ	0.03	2.12	<LOQ	0.004	2.96
	EtOH 50%	24h	[μg/L]	0.41	<LOQ	7.00	0.08	<LOQ	0.25	<LOQ	0.05	0.71	<LOQ	217	<LOQ	<LOQ	0.08	0.06	<LOQ	27.44	<LOQ	0.001	2.56
	EtOH 50%	10d	[μg/L]	<LOQ	<LOQ	19.85	0.23	0.57	0.98	41.94	0.13	3.37	<LOQ	553	<LOQ	<LOQ	0.09	0.09	<LOQ	<LOQ	<LOQ	0.002	3.61
	H2O	24h	[μg/L]	<LOQ	<LOQ	<LOQ	<LOQ	<LOQ	<LOQ	<LOQ	<LOQ	<LOQ	<LOQ	134	<LOQ	<LOQ	0.01	<LOQ	0.08	<LOQ	<LOQ	<LOQ	0.75
	H2O	10d	[μg/L]	<LOQ	0.085	34.50	0.03	<LOQ	<LOQ	<LOQ	0.01	0.54	<LOQ	348	0.01	<LOQ	0.02	<LOQ	0.02	1.35	0.001	0.002	0.23
EPDM (2)	AcAc	24h	[μg/L]	0.04	<LOQ	63.62	0.06	<LOQ	<LOQ	22.23	<LOQ	1.05	2.26	2401	0.01	0.02	0.02	0.15	0.14	2.41	<LOQ	0.04	0.38
	AcAc	10d	[μg/L]	<LOQ	<LOQ	137.93	0.10	2.11	0.97	44.42	<LOQ	11.86	1.43	2837	<LOQ	<LOQ	<LOQ	<LOQ	0.03	2.17	<LOQ	0.04	0.34
	EtOH 10%	24h	[μg/L]	<LOQ	<LOQ	<LOQ	<LOQ	<LOQ	<LOQ	<LOQ	<LOQ	<LOQ	<LOQ	182	<LOQ	<LOQ	<LOQ	<LOQ	0.07	0.59	<LOQ	0.06	<LOQ
	EtOH 10%	10d	[μg/L]	<LOQ	<LOQ	<LOQ	<LOQ	<LOQ	<LOQ	<LOQ	<LOQ	<LOQ	<LOQ	917	<LOQ	<LOQ	0.01	<LOQ	0.60	<LOQ	<LOQ	0.11	<LOQ
	EtOH 50%	24h	[μg/L]	0.04	<LOQ	3.74	0.14	<LOQ	<LOQ	<LOQ	<LOQ	0.93	<LOQ	410	<LOQ	<LOQ	<LOQ	0.04	0.32	17.57	<LOQ	0.05	<LOQ
	EtOH 50%	10d	[μg/L]	<LOQ	<LOQ	7.75	0.24	0.57	1.15	41.06	0.12	0.46	<LOQ	919	<LOQ	<LOQ	0.09	0.07	0.51	<LOQ	<LOQ	0.10	3.03
	H2O	24h	[μg/L]	<LOQ	<LOQ	6.98	<LOQ	<LOQ	<LOQ	<LOQ	<LOQ	<LOQ	<LOQ	127	<LOQ	<LOQ	<LOQ	<LOQ	<LOQ	0.73	<LOQ	0.01	0.02
	H2O	10d	[μg/L]	<LOQ	0.07	32.27	0.01	<LOQ	<LOQ	<LOQ	<LOQ	0.04	<LOQ	323	0.005	<LOQ	<LOQ	<LOQ	0.017	1.72	<LOQ	0.17	<LOQ
NR/SBR (3)	AcAc	24h	[μg/L]	0.01	<LOQ	47.59	0.09	<LOQ	0.29	14.45	0.07	2.89	<LOQ	4882	0.02	<LOQ	0.34	<LOQ	0.02	0.18	<LOQ	0.02	149.27
	AcAc	10d	[μg/L]	<LOQ	<LOQ	386.99	0.68	1.12	5.16	87.02	<LOQ	10.86	<LOQ	22304	0.12	<LOQ	0.62	<LOQ	0.04	2.37	<LOQ	0.09	558.99
	EtOH 10%	24h	[μg/L]	<LOQ	<LOQ	<LOQ	<LOQ	<LOQ	<LOQ	<LOQ	0.05	<LOQ	<LOQ	1038	<LOQ	<LOQ	<LOQ	<LOQ	<LOQ	<LOQ	<LOQ	0.05	8.93
	EtOH 10%	10d	[μg/L]	<LOQ	<LOQ	<LOQ	<LOQ	<LOQ	1.97	<LOQ	<LOQ	<LOQ	<LOQ	972	<LOQ	<LOQ	0.05	<LOQ	0.03	<LOQ	<LOQ	0.12	8.30
	EtOH 50%	24h	[μg/L]	<LOQ	<LOQ	<LOQ	<LOQ	<LOQ	1.86	<LOQ	0.52	3.47	8.97	2779	<LOQ	<LOQ	0.29	<LOQ	<LOQ	<LOQ	0.02	0.06	108.70
	EtOH 50%	10d	[μg/L]	0.21	<LOQ	<LOQ	0.08	<LOQ	3.58	30.93	1.42	6.10	10.65	4693	<LOQ	<LOQ	0.18	<LOQ	<LOQ	89.00	0.03	0.10	89.47
	H2O	24h	[μg/L]	<LOQ	<LOQ	<LOQ	<LOQ	<LOQ	<LOQ	<LOQ	<LOQ	<LOQ	<LOQ	1314	<LOQ	<LOQ	0.19	<LOQ	<LOQ	<LOQ	<LOQ	0.06	7.89
	H2O	10d	[μg/L]	<LOQ	0.03	0.21	<LOQ	<LOQ	3.61	<LOQ	0.01	<LOQ	<LOQ	2029	0.00	<LOQ	0.03	<LOQ	0.00	0.17	0.00	0.20	11.69

Table S3. Levels of Elements in food simulants (continued)

Sample type (sample #)	Food simulant	Migration time	unit	Li	Be	Al	V	Cr	Mn	Fe	Co	Ni	Cu	Zn	As	Mo	Cd	Sn	Sb	Ba	Hg	Tl	Pb
NBR (4)	AcAc	24h	[µg/L]	0.51	<LOQ	288.75	0.96	1.60	20.35	100.67	0.06	2.46	<LOQ	137029	0.12	0.01	0.45	0.22	0.12	8.42	<LOQ	0.057	38.84
	AcAc	10d	[µg/L]	2.07	0.19	1265.49	3.27	6.95	58.68	318.54	<LOQ	8.61	5.59	333017	0.36	<LOQ	0.52	0.17	0.12	19.07	<LOQ	0.149	54.73
	EtOH 10%	24h	[µg/L]	<LOQ	<LOQ	<LOQ	<LOQ	<LOQ	<LOQ	<LOQ	<LOQ	<LOQ	<LOQ	1639	<LOQ	<LOQ	<LOQ	<LOQ	<LOQ	0.40	<LOQ	0.005	2.03
	EtOH 10%	10d	[µg/L]	<LOQ	<LOQ	<LOQ	<LOQ	<LOQ	<LOQ	<LOQ	<LOQ	<LOQ	<LOQ	3861	<LOQ	<LOQ	0.36	0.07	0.10	<LOQ	<LOQ	0.011	4.47
	EtOH 50%	24h	[µg/L]	2.37	<LOQ	<LOQ	<LOQ	<LOQ	0.40	<LOQ	0.10	0.69	<LOQ	2563	<LOQ	<LOQ	0.42	<LOQ	<LOQ	14.23	<LOQ	0.006	30.07
	EtOH 50%	10d	[µg/L]	0.40	<LOQ	<LOQ	0.13	<LOQ	1.88	52.11	1.21	7.75	5.33	9885	<LOQ	<LOQ	1.08	<LOQ	0.08	<LOQ	<LOQ	0.021	57.89
	H2O	24h	[µg/L]	<LOQ	<LOQ	<LOQ	<LOQ	<LOQ	<LOQ	<LOQ	<LOQ	<LOQ	<LOQ	1631	<LOQ	<LOQ	0.12	<LOQ	<LOQ	<LOQ	<LOQ	0.004	2.89
	H2O	10d	[µg/L]	0.00	0.02	<LOQ	<LOQ	<LOQ	0.22	<LOQ	<LOQ	<LOQ	<LOQ	838	0.00	<LOQ	0.02	<LOQ	0.01	0.02	0.00	0.003	0.61
TPE-S (5)*	H2O	24h	[µg/L]	<LOQ	<LOQ	45.59	<LOQ	<LOQ	<LOQ	<LOQ	<LOQ	<LOQ	<LOQ	9	<LOQ	<LOQ	<LOQ	<LOQ	<LOQ	1.34	<LOQ	0.001	0.02
TPE-S (6)	AcAc	24h	[µg/L]	<LOQ	<LOQ	13.67	0.01	<LOQ	0.48	9.09	0.03	4.07	2.46	95	0.01	0.01	0.01	<LOQ	0.03	0.51	<LOQ	0.000	0.33
	AcAc	10d	[µg/L]	<LOQ	<LOQ	15.09	0.02	<LOQ	2.26	23.39	<LOQ	2.78	11.01	342	<LOQ	<LOQ	<LOQ	<LOQ	0.05	0.55	<LOQ	0.001	0.23
	EtOH 10%	24h	[µg/L]	0.20	<LOQ	0.32	<LOQ	<LOQ	<LOQ	<LOQ	0.11	0.70	<LOQ	46	<LOQ	<LOQ	<LOQ	<LOQ	0.03	<LOQ	0.02	<LOQ	<LOQ
	EtOH 10%	10d	[µg/L]	<LOQ	<LOQ	5.76	<LOQ	<LOQ	<LOQ	<LOQ	<LOQ	2.86	<LOQ	45	<LOQ	<LOQ	0.004	0.09	0.04	<LOQ	<LOQ	<LOQ	0.06
	EtOH 50%	24h	[µg/L]	0.09	<LOQ	<LOQ	<LOQ	<LOQ	<LOQ	<LOQ	<LOQ	2.35	2.40	133	<LOQ	<LOQ	<LOQ	<LOQ	<LOQ	<LOQ	<LOQ	<LOQ	<LOQ
	EtOH 50%	10d	[µg/L]	<LOQ	<LOQ	4.17	0.10	<LOQ	0.92	30.93	0.23	2.04	2.00	1433	<LOQ	<LOQ	0.88	0.08	<LOQ	46.13	0.02	0.003	40.11
	H2O	24h	[µg/L]	<LOQ	<LOQ	<LOQ	<LOQ	<LOQ	<LOQ	<LOQ	<LOQ	<LOQ	<LOQ	243	<LOQ	<LOQ	<LOQ	<LOQ	<LOQ	<LOQ	<LOQ	<LOQ	0.06
	H2O	10d	[µg/L]	<LOQ	0.07	9.20	<LOQ	<LOQ	<LOQ	<LOQ	<LOQ	1.99	<LOQ	94	0.01	<LOQ	0.003	<LOQ	0.01	0.59	<LOQ	0.001	0.51
NBR (7)	AcAc	24h	[µg/L]	1.86	<LOQ	224.70	3.73	2.46	264.11	423.46	0.48	18.41	<LOQ	143810	0.52	0.22	0.02	<LOQ	0.11	11.27	<LOQ	0.051	8.26
	AcAc	10d	[µg/L]	5.19	0.25	848.44	4.24	5.05	1068.35	1601.92	0.66	114.42	2.81	291942	1.70	0.52	0.05	<LOQ	0.35	46.70	<LOQ	0.117	29.09
	EtOH 10%	24h	[µg/L]	0.29	<LOQ	8.66	<LOQ	<LOQ	<LOQ	<LOQ	0.07	<LOQ	<LOQ	202	<LOQ	<LOQ	<LOQ	<LOQ	<LOQ	<LOQ	<LOQ	0.006	<LOQ
	EtOH 10%	10d	[µg/L]	0.67	<LOQ	12.08	0.13	<LOQ	<LOQ	<LOQ	<LOQ	<LOQ	<LOQ	192	<LOQ	<LOQ	0.00	<LOQ	0.03	<LOQ	<LOQ	0.010	0.30
	EtOH 50%	24h	[µg/L]	0.14	<LOQ	<LOQ	<LOQ	<LOQ	<LOQ	<LOQ	0.34	20.95	2.31	1062	<LOQ	<LOQ	0.02	<LOQ	<LOQ	16.81	<LOQ	0.009	8.62
	EtOH 50%	10d	[µg/L]	1.22	<LOQ	<LOQ	0.09	0.54	1.03	40.31	1.00	47.21	1.98	2469	<LOQ	0.08	0.79	<LOQ	<LOQ	92.97	0.03	0.015	14.21
	H2O	24h	[µg/L]	0.05	<LOQ	28.19	0.11	<LOQ	1.68	<LOQ	<LOQ	<LOQ	<LOQ	457	<LOQ	<LOQ	<LOQ	<LOQ	<LOQ	<LOQ	<LOQ	0.010	0.83
	H2O	10d	[µg/L]	0.98	0.03	7.98	1.29	<LOQ	<LOQ	<LOQ	<LOQ	0.01	<LOQ	866	0.08	0.08	<LOQ	<LOQ	0.08	0.93	<LOQ	0.012	1.12
PUR (8)*	AcAc	24h	[µg/L]	<LOQ	<LOQ	8.04	<LOQ	<LOQ	<LOQ	3.95	0.07	3.31	<LOQ	<LOQ	0.01	<LOQ	<LOQ	7.65	<LOQ	<LOQ	<LOQ	<LOQ	0.03
	H2O	24h	[µg/L]	<LOQ	<LOQ	14.18	<LOQ	<LOQ	<LOQ	<LOQ	<LOQ	<LOQ	<LOQ	72	<LOQ	<LOQ	<LOQ	1.99	<LOQ	<LOQ	<LOQ	0.001	0.06

Table S3. Levels of Elements in food simulants (continued)

Sample type (sample #)	Food simulant	Migration time	unit	Li	Be	Al	V	Cr	Mn	Fe	Co	Ni	Cu	Zn	As	Mo	Cd	Sn	Sb	Ba	Hg	Tl	Pb
(8)**	AcAc	24h	[µg/L]	<LOQ	<LOQ	6.85	<LOQ	<LOQ	<LOQ	8.84	<LOQ	0.41	<LOQ	<LOQ	<LOQ	<LOQ	<LOQ	<LOQ	<LOQ	<LOQ	<LOQ	<LOQ	<LOQ
	AcAc	10d	[µg/L]	<LOQ	<LOQ	5.35	<LOQ	<LOQ	<LOQ	15.83	<LOQ	<LOQ	<LOQ	106	<LOQ	<LOQ	<LOQ	0.13	0.02	<LOQ	<LOQ	0.001	0.03
	EtOH 10%	24h	[µg/L]	0.22	<LOQ	<LOQ	<LOQ	<LOQ	<LOQ	<LOQ	<LOQ	<LOQ	<LOQ	109	<LOQ	<LOQ	<LOQ	<LOQ	<LOQ	<LOQ	<LOQ	<LOQ	<LOQ
	EtOH 10%	10d	[µg/L]	<LOQ	<LOQ	<LOQ	<LOQ	<LOQ	<LOQ	<LOQ	<LOQ	<LOQ	<LOQ	94	<LOQ	<LOQ	<LOQ	<LOQ	<LOQ	<LOQ	<LOQ	0.001	0.02
	EtOH 50%	24h	[µg/L]	0.24	<LOQ	<LOQ	<LOQ	<LOQ	<LOQ	<LOQ	<LOQ	<LOQ	<LOQ	129	<LOQ	<LOQ	<LOQ	<LOQ	<LOQ	11.20	<LOQ	0.001	<LOQ
	EtOH 50%	10d	[µg/L]	1.00	<LOQ	10.76	0.10	<LOQ	0.70	26.12	1.25	12.53	3.99	412	<LOQ	0.08	<LOQ	<LOQ	<LOQ	<LOQ	<LOQ	0.004	11.38
	H2O	24h	[µg/L]	<LOQ	<LOQ	<LOQ	<LOQ	<LOQ	<LOQ	<LOQ	<LOQ	<LOQ	<LOQ	135	<LOQ	<LOQ	<LOQ	<LOQ	<LOQ	<LOQ	<LOQ	<LOQ	<LOQ
	H2O	10d	[µg/L]	<LOQ	0.01	1.98	<LOQ	<LOQ	<LOQ	<LOQ	<LOQ	<LOQ	<LOQ	72	<LOQ	<LOQ	<LOQ	<LOQ	<LOQ	0.02	<LOQ	<LOQ	<LOQ
9	AcAc	24h	[µg/L]	<LOQ	<LOQ	221.09	0.17	<LOQ	1.30	14.76	<LOQ	0.47	<LOQ	44568	0.09	<LOQ	<LOQ	<LOQ	0.03	46.59	<LOQ	0.022	<LOQ
	AcAc	10d	[µg/L]	<LOQ	<LOQ	290.37	0.37	<LOQ	2.97	62.71	<LOQ	<LOQ	<LOQ	108417	0.28	0.13	<LOQ	<LOQ	0.06	83.74	<LOQ	0.076	0.08
	EtOH 10%	24h	[µg/L]	0.24	<LOQ	<LOQ	<LOQ	<LOQ	<LOQ	<LOQ	<LOQ	<LOQ	<LOQ	1323	<LOQ	<LOQ	<LOQ	<LOQ	<LOQ	15.97	<LOQ	0.021	<LOQ
	EtOH 10%	10d	[µg/L]	<LOQ	<LOQ	<LOQ	<LOQ	<LOQ	2.97	<LOQ	<LOQ	<LOQ	<LOQ	5539	<LOQ	<LOQ	<LOQ	<LOQ	<LOQ	18.41	<LOQ	0.060	<LOQ
	EtOH 50%	24h	[µg/L]	<LOQ	<LOQ	<LOQ	<LOQ	<LOQ	1.60	<LOQ	<LOQ	<LOQ	<LOQ	3626	<LOQ	<LOQ	0.02	<LOQ	<LOQ	<LOQ	<LOQ	0.038	0.17
	EtOH 50%	10d	[µg/L]	1.12	<LOQ	39.07	0.15	<LOQ	7.87	55.53	1.49	30.68	5.03	9621	<LOQ	0.16	<LOQ	0.21	<LOQ	91.33	<LOQ	0.089	3.82
	H2O	24h	[µg/L]	<LOQ	<LOQ	3.38	<LOQ	<LOQ	<LOQ	<LOQ	<LOQ	<LOQ	<LOQ	333	<LOQ	<LOQ	<LOQ	<LOQ	<LOQ	2.51	<LOQ	0.003	<LOQ
	H2O	10d	[µg/L]	<LOQ	0.02	3.66	<LOQ	<LOQ	1.10	<LOQ	<LOQ	<LOQ	<LOQ	6757	<LOQ	<LOQ	<LOQ	<LOQ	0.03	18.63	<LOQ	0.053	<LOQ
10	AcAc	24h	[µg/L]	34.86	0.11	288.65	3.71	7.00	46.37	567.53	0.33	5.32	<LOQ	91447	0.79	0.32	0.19	<LOQ	0.08	4.93	<LOQ	0.194	40.02
	AcAc	10d	[µg/L]	142.55	0.89	1234.87	13.63	26.76	219.53	5894.63	1.34	18.86	3.95	242867	3.62	2.54	0.50	<LOQ	0.55	28.21	0.06	0.707	204.29
	EtOH 10%	24h	[µg/L]	0.82	<LOQ	<LOQ	<LOQ	<LOQ	<LOQ	<LOQ	0.27	2.66	<LOQ	785	0.13	<LOQ	<LOQ	<LOQ	<LOQ	<LOQ	<LOQ	0.067	26.18
	EtOH 10%	10d	[µg/L]	6.09	<LOQ	<LOQ	<LOQ	<LOQ	<LOQ	<LOQ	<LOQ	1.05	<LOQ	2219	0.25	<LOQ	0.21	<LOQ	<LOQ	<LOQ	<LOQ	0.099	89.86
	EtOH 50%	24h	[µg/L]	9.02	<LOQ	<LOQ	<LOQ	<LOQ	<LOQ	<LOQ	0.69	11.92	7.89	3651	0.22	<LOQ	0.33	<LOQ	<LOQ	<LOQ	<LOQ	0.027	232.86
	EtOH 50%	10d	[µg/L]	24.43	<LOQ	15.51	<LOQ	2.90	5.32	63.21	4.27	19.71	34.36	5575	0.47	0.22	0.83	0.27	<LOQ	160.93	<LOQ	0.123	540.52
	H2O	24h	[µg/L]	0.85	<LOQ	<LOQ	<LOQ	<LOQ	<LOQ	<LOQ	<LOQ	<LOQ	<LOQ	745	0.58	<LOQ	0.06	<LOQ	<LOQ	<LOQ	<LOQ	0.018	15.64
	H2O	10d	[µg/L]	1.87	0.02	<LOQ	<LOQ	<LOQ	<LOQ	<LOQ	0.01	<LOQ	<LOQ	1111	0.22	<LOQ	0.10	<LOQ	<LOQ	<LOQ	<LOQ	0.057	17.75

*limited amount of sample available **Elastomer was a colored version (blue) original whitish sample; LOQ: limit of quantification

Table S4. Limits of quantification (LOQ) in µg/l for elements in different matrices as determined by blank value method

Element	water	3% acetic acid	10 % ethanol	50 % ethanol
**Li**	0.085	0.036	0.20	0.25
**Be**	0.048	0.13	0.057	0.093
**Al**	3.33	4.04	6.06	7.49
**V**	0.011	0.026	0.030	0.080
**Cr**	1.34	0.58	1.46	0.55
**Mn**	0.52	1.21	0.57	0.15
**Fe**	33.1	5.12	1.64	12.28
**Co**	0.13	0.057	0.042	0.04
**Ni**	0.83	0.28	0.85	0.27
**Cu**	0.73	4.45	1.97	1.41
**Zn**	38.2	139	7.94	5.92
**As**	0.0057	0.0059	0.029	0.14
**Mo**	0.11	0.025	0.032	0.10
**Cd**	0.0031	0.013	0.022	0.048
**Sn**	0.34	0.38	0.16	0.10
**Sb**	0.021	0.0029	0.04	0.072
**Ba**	0.53	0.18	0.16	58.07
**Hg**	0.019	0.018	0.014	0.033
**Tl**	0.00052	0.00025	0.00057	0.00047
**Pb**	0.081	0.069	0.38	0.087

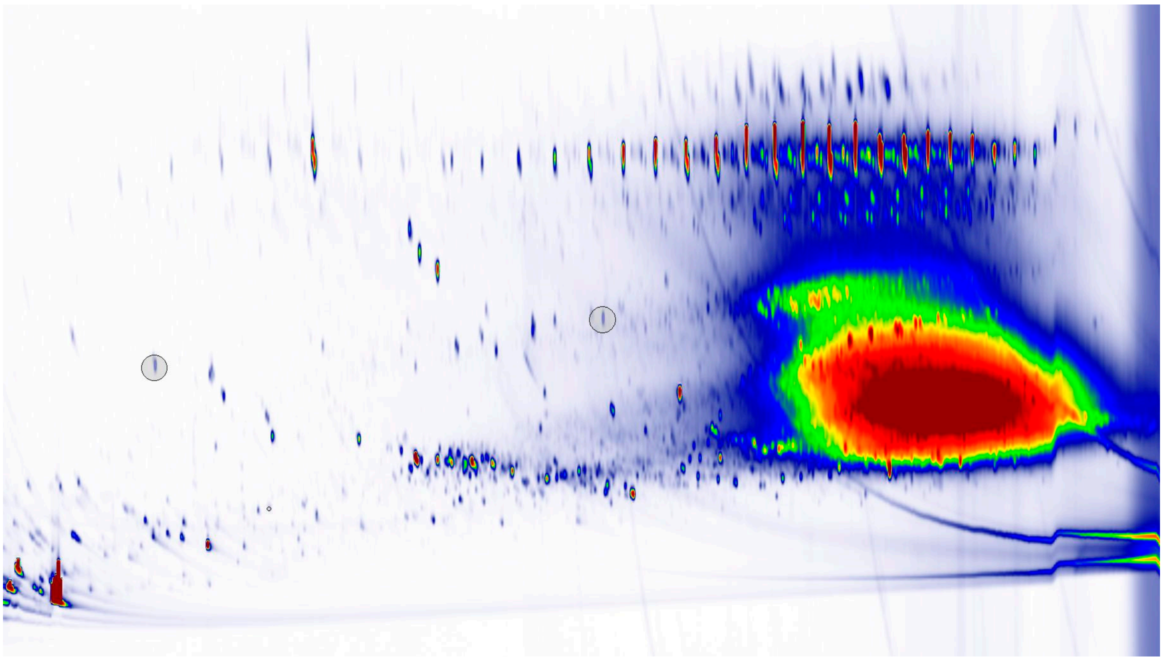

Figure S1: GCxGC-FID chromatogram of the extract from IIR (sample #1).Axes as labled in figure 2.

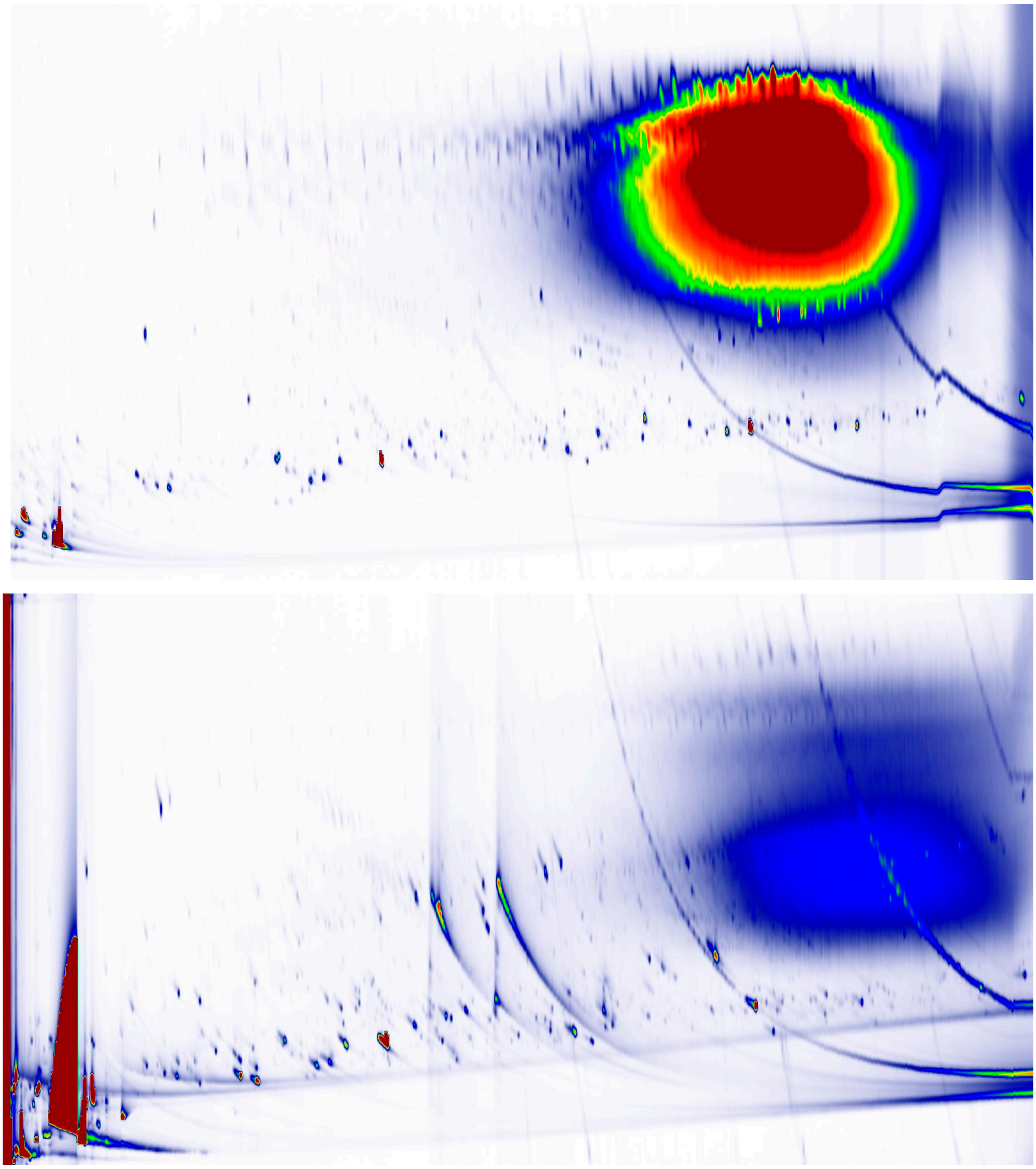

Figure S2: GCxGC-FID chromatograms of the extract from EPDM (sample #2). The top chromatogram is the extract the bottom is the extract after removal of MOSH by LC. Axes as labled in figure 2.

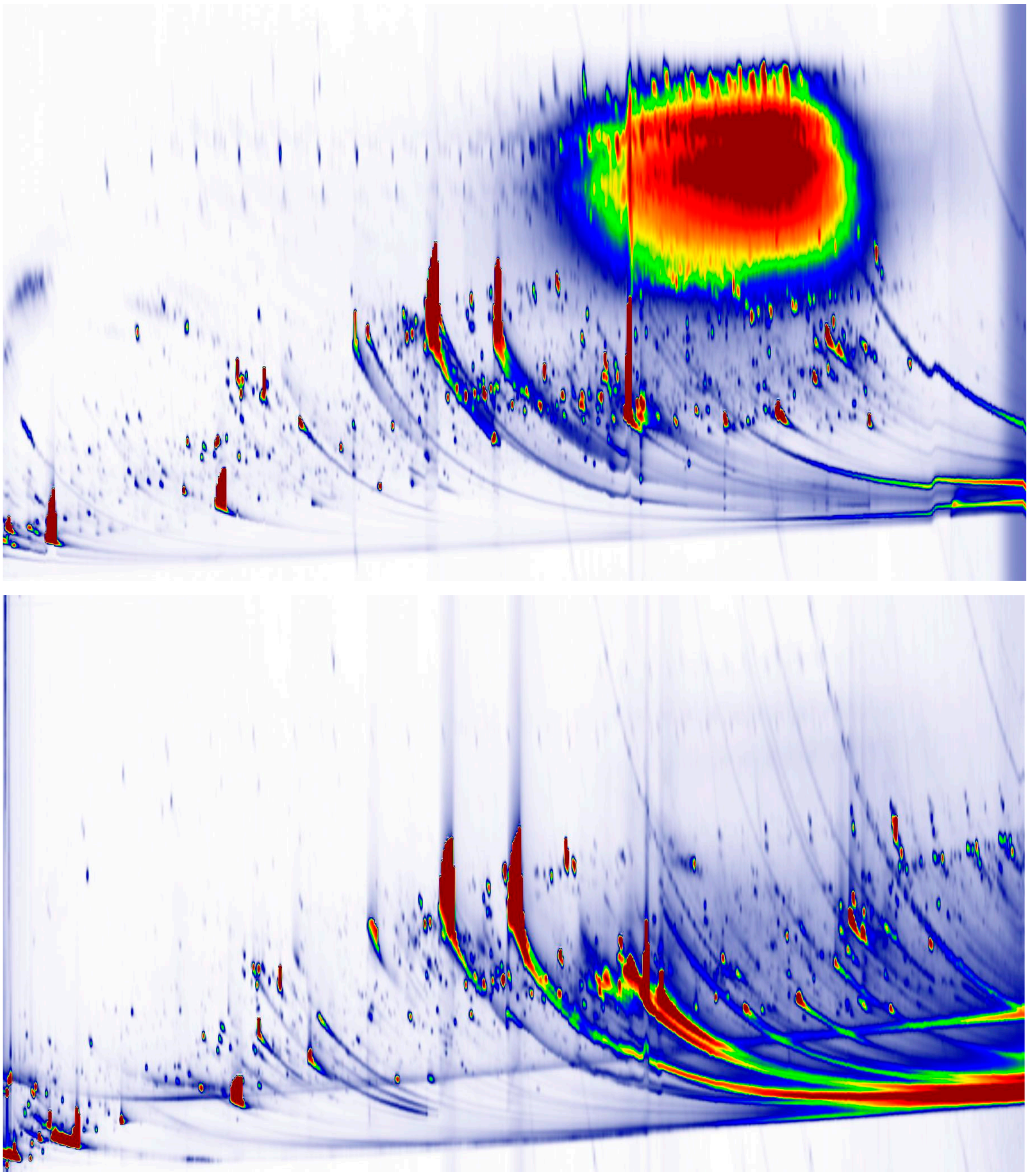

Figure S3: GCxGC-FID chromatograms of the extract from NR/SBR (sample #3). The top chromatogram is the extract the bottom is the extract after removal of MOSH by LC. Axes as labled in figure 2.

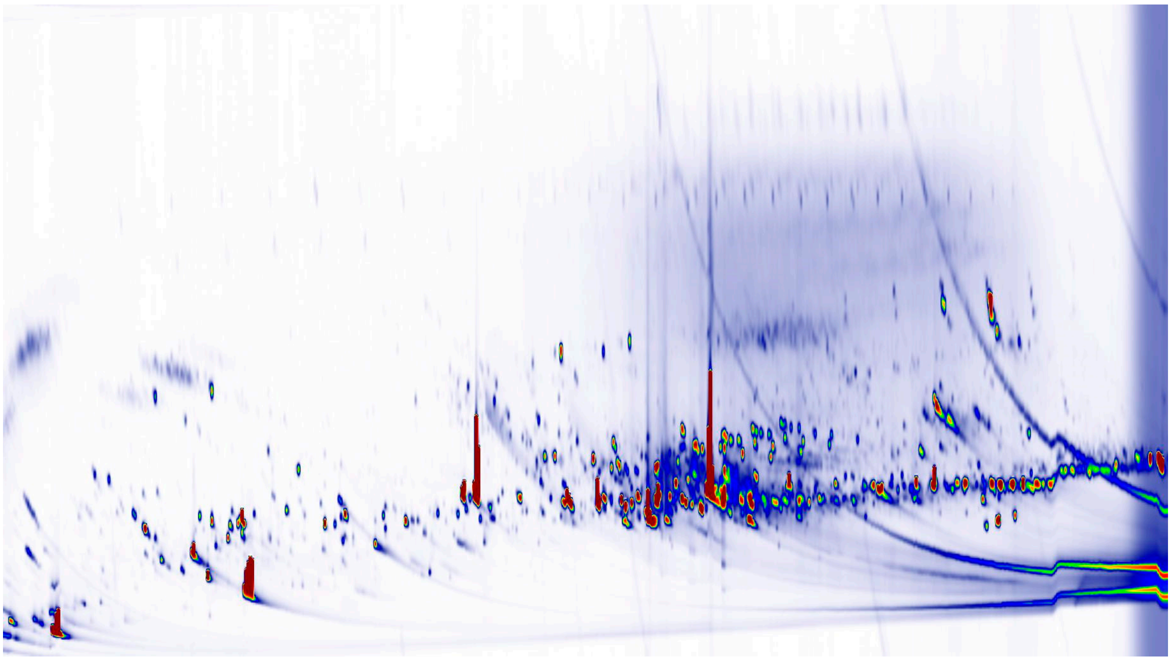

Figure S4: GCxGC-FID chromatogram of the extract from NBR (sample #4). Axes as labled in figure 2.

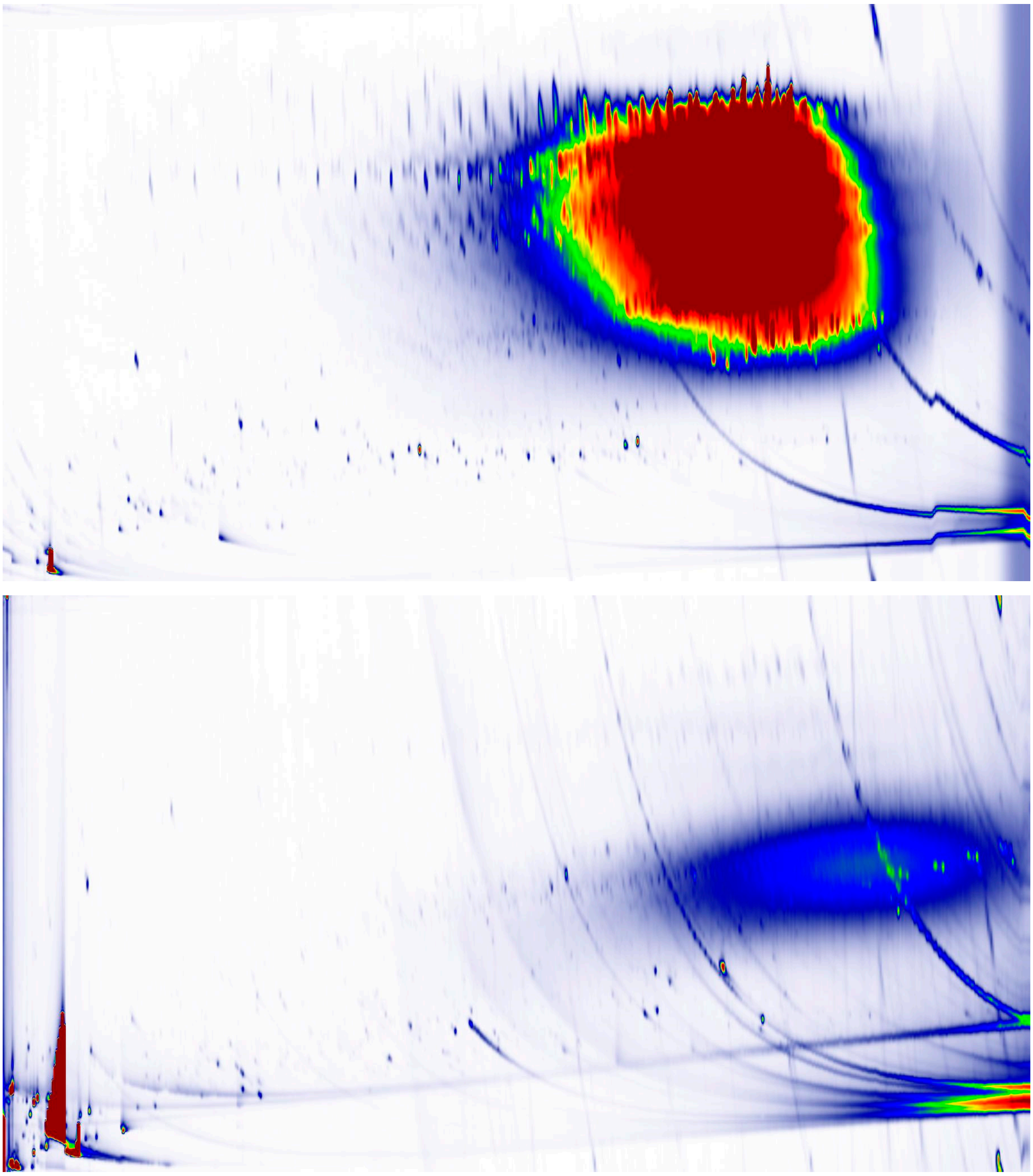

Figure S5: GCxGC-FID chromatograms of the extract from TPE (SEBS) (sample #5). The top chromatogram is the extract the bottom is the extract after removal of MOSH by LC. Axes as labled in figure 2.

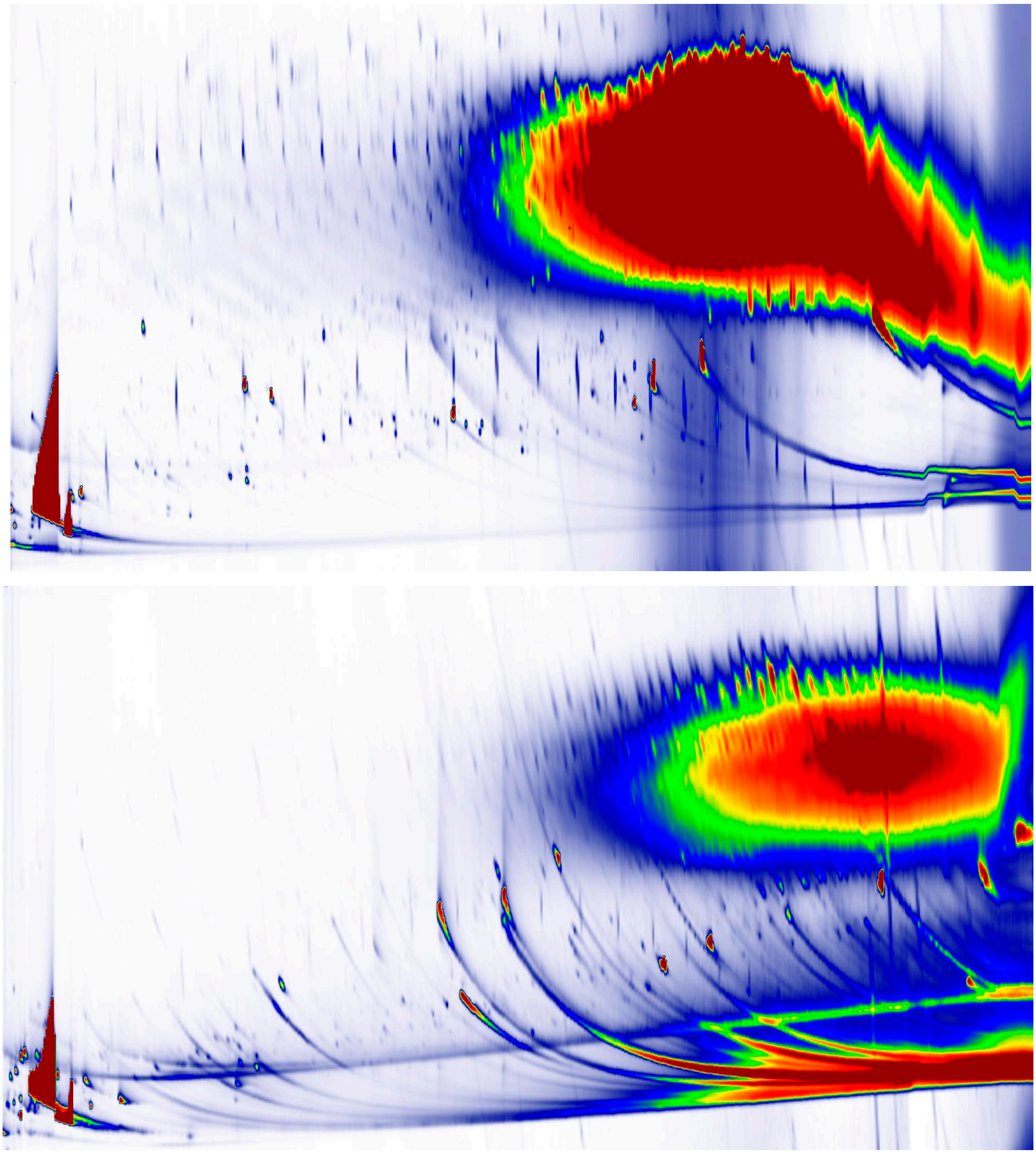

Figure S6: GCxGC-FID chromatograms of the extract from TPE (SEBS) (sample #6). The top chromatogram is the extract the bottom is the extract after removal of MOSH by LC. Axes as labled in figure 2.

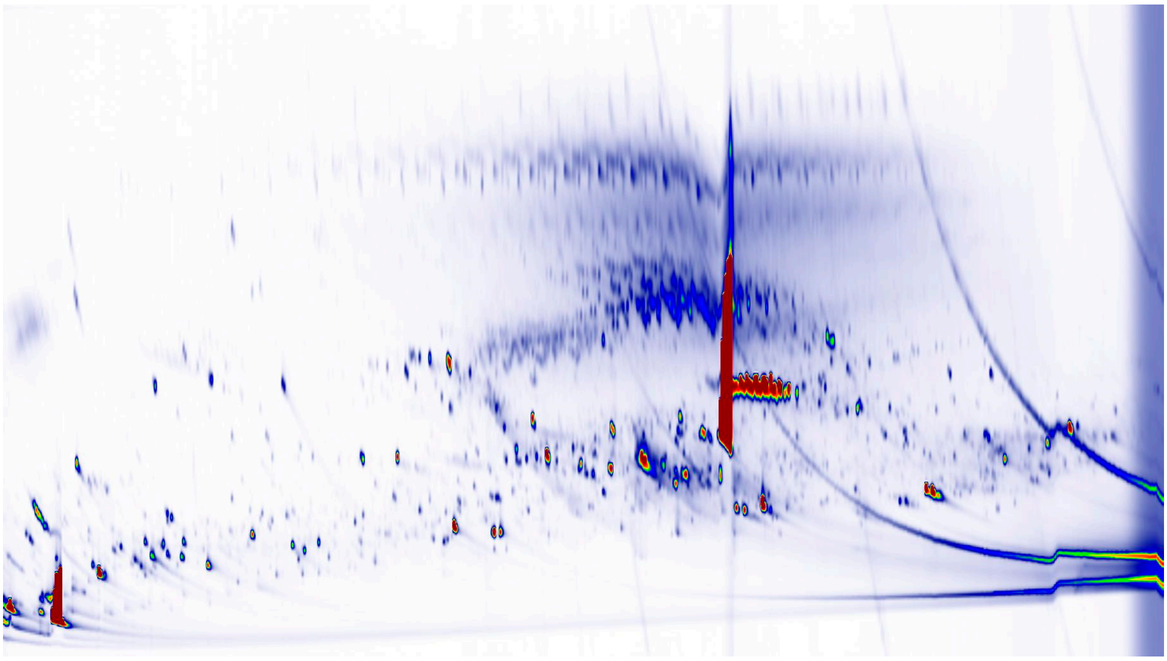

Figure S7: GCxGC chromatogram of the extract from NBR (sample #7). Axes as labled in figure 2.

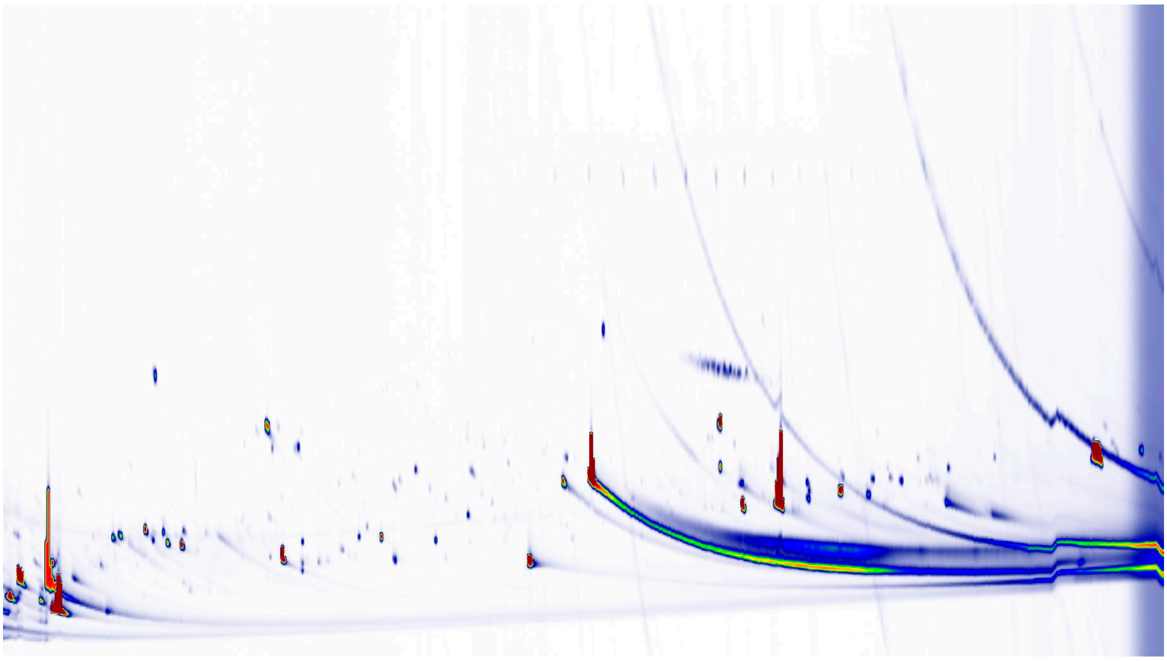

Figure S8: GCxGC-FID chromatogram of the extract from PUR (sample #8). Axes as labled in figure 2.

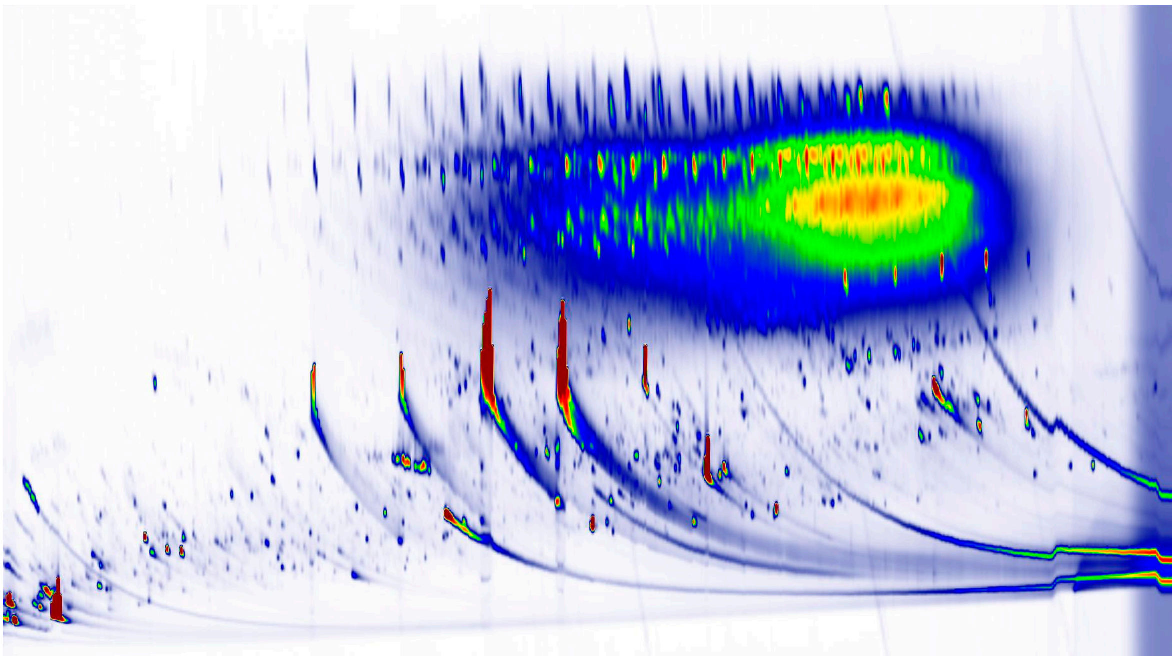

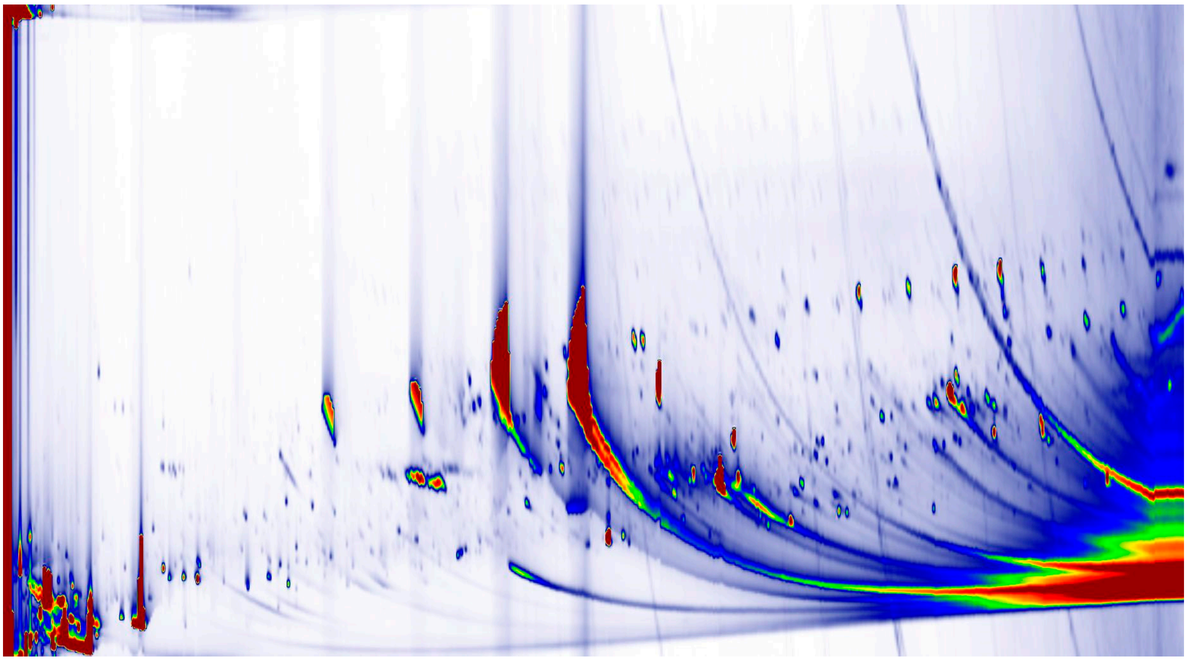

Figure S9: GCxGC-FID chromatograms of the extract from NR/SBR (sample #9). The top chromatogram is the extract the bottom is the extract after removal of MOSH by LC. Axes as labled in figure 2.

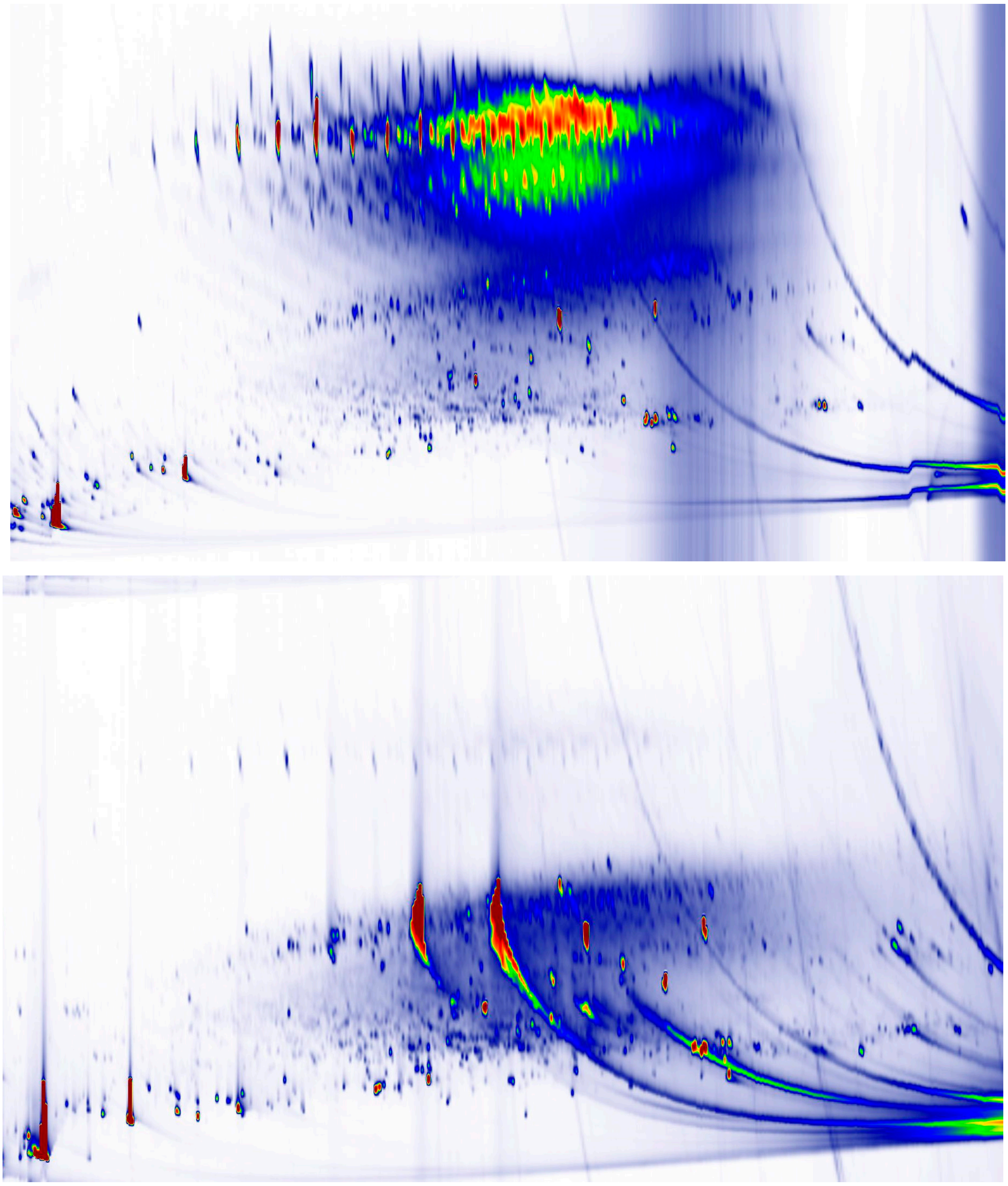

Figure S10: GCxGC-FID chromatograms of the extract from SBS/NR (sample #10). The top chromatogram is the extract the bottom is the extract after removal of MOSH by LC.

Only sample #10was re-run after removal of MOSH and MOAH by LC. The results are shown in figure S11. In this sample it was thought that peaks could be covered by the MOAH signals. *Axes as labled in figure 2.*

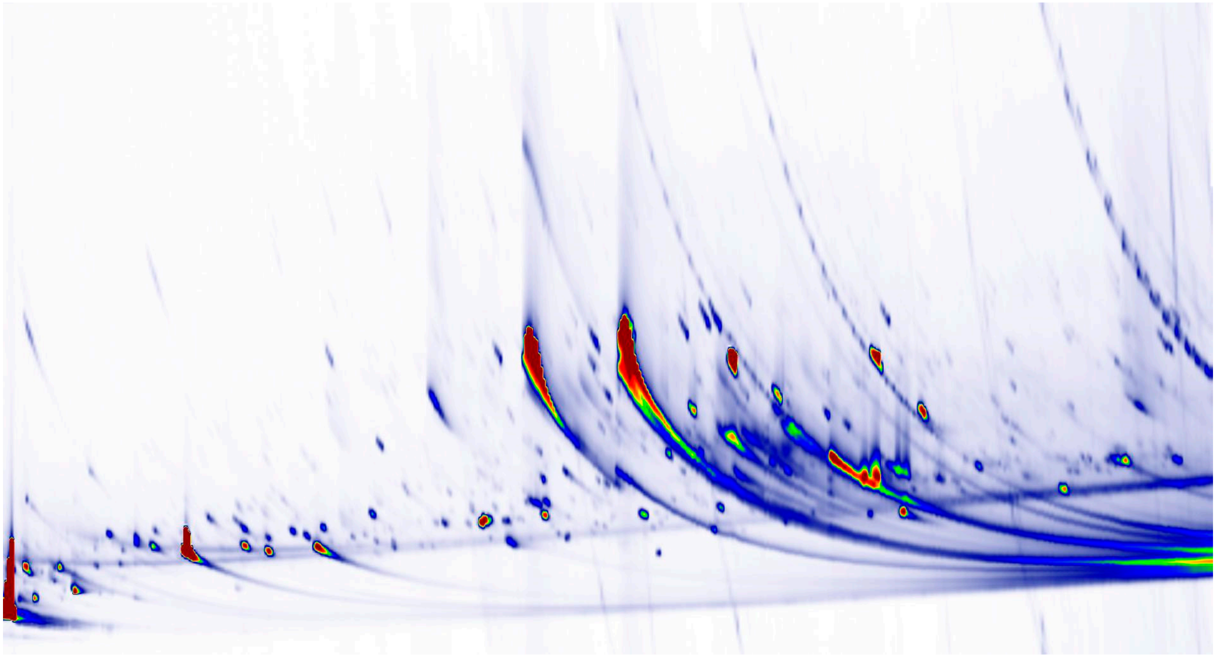

Figure S11: GCxGC-FID chromatogram of the extract from SBS/NR (sample #10) after the removal of MOSH and MOAH by LC. Axes as labled in figure 2.
